# Interventions Involving Caregivers for Children and Adolescents Following Traumatic Events: A Systematic Review and Meta-Analysis

**DOI:** 10.1007/s10567-022-00415-2

**Published:** 2022-09-26

**Authors:** Katharina Szota, Katharina Louisa Schulte, Hanna Christiansen

**Affiliations:** Department of Psychology, Philipps-University of Marburg, Gutenbergstr. 18, 35032 Marburg, Hesse Germany

**Keywords:** Child and Adolescents, Trauma-related Psychopathology, Posttraumatic Stress Disorder, PTSD, Treatment, Intervention, Caregiver involvement, Parents

## Abstract

Although treatment guidelines recommend interventions entailing caregiver involvement for children and adolescents following traumatic experiences, evidence on their effectiveness is inconsistent. The present systematic review and meta-analysis considered possible moderators of their effectiveness. Method. Eligible studies were (quasi-)randomized controlled trials and efficacy trials published in English or German with participants up to the age of 21 years presenting symptoms of mental disorders due to traumatic experiences. The effectiveness of interventions entailing any kind and extent of caregiver involvement had to be investigated by applying evaluated instruments. PubMed, PsycINFO, ERIC, COCHRANE and PSYNDEX were searched. Results. A total of 33 studies with 36 independent samples were retrieved. Child- and parent-reports on PTSD, depression, anxiety, ADHD, internalizing, externalizing symptoms and behavior problems were analyzed where available. The pooled effect size is significant and robust at post-treatment for child-reported PTSD, *g* = − 0.34 (95% *CI* = − 0.53; − 0.14), parent-reported PTSD, *g* = − 0.41 (95% *CI* = − 0.71; − 0.11), child-reported depression, *g* = − 0.29 (95% *CI* = − 0.46; − 0.11), child-reported anxiety, *g* = − 0.25 (95% *CI* = − 0.42; − 0.08), and parent-reported internalizing symptoms, *g* = − 0.27 (95% *CI* = − 0.47; − 0.07). Female sex and fulfilling diagnostic criteria appeared as potential moderators. The only significant effect size at follow-up is found for child-reported PTSD symptoms 12 months post-treatment, *g* = − 0.37 (95% *CI* = − 0.67; − 0.07). Conclusions. Interventions entailing caregiver involvement revealed greater symptom reductions than control conditions. Determinants of their effectiveness should be examined further.

**Supplementary Information:**

The online version contains supplementary material available at 10.1007/s10567-022-00415-2.

## Background

Traumatic experiences affect many children and adolescents with prevalence rates between 31.1% (Lewis et al., 2019) and 56.1% (Landolt et al., 2013). They are associated with a higher risk for several mental disorders (Asselmann et al., 2018; Lewis et al., 2019). Posttraumatic stress disorders (PTSD) are developed by 15.9% of trauma-exposed children and adolescents (Alisic et al., 2014) and the majority has comorbid diagnoses (Basu et al., 2020; Perkonigg et al., 2000). Other relevant outcomes after trauma exposure are anxiety and depressive disorders (Asselmann et al., 2018; Lewis et al., 2019).

Current meta-analyses show that there are effective psychological treatments for children and adolescents affected by traumatic events (Gutermann et al., 2016; Morina et al., 2016). Practitioners and researchers argue that the involvement of caregivers (e.g. parents, grandparents, foster parents, youth welfare caregivers) is an important component of those treatments resulting in better outcomes (Kar, 2009; Stallard, 2006). Interventions involving caregivers can improve caregivers’ ability to support their children during recovery (Cohen et al., 2004; Tutus et al., 2019). Two studies show that caregiver behavior during the trauma processing sessions within the trauma-focused cognitive behavior therapy (Tf-CBT) by Cohen et al. (2016) is associated with in-session child distress (Canale et al., 2022) and predicts symptom change in youth (Yasinski et al., 2016). The latest treatment guidelines for PTSD in childhood and adolescence recommend interventions involving caregivers, as published by the National Institute for Health and Care Excellence [NICE] (2018), and the German-language Society for Psychotraumatology (Deutschsprachige Gesellschaft für Psychotraumatologie [DeGPT]) (Schäfer et al., 2019).

Several meta-analyses investigated whether interventions involving caregivers are more beneficial than interventions with no caregiver involvement. One meta-analysis that included both controlled and uncontrolled studies detected a positive effect of caregiver involvement on PTSD symptom reduction (Gutermann et al., 2016). The majority of investigated studies (56%) included treatments that were identified as CBT; further, of the 84 CBT treatment conditions, 46 (54%) were investigated with uncontrolled conditions. However, this moderating effect was not replicated in their follow-up analyses (Gutermann et al., 2017). Note that those studies without control groups that only assess whether symptom levels drop during and after treatment cannot ensure that any improvements are directly attributable to treatment effects and not to external factors or symptom reductions over time. Still, these results are in line with those of a meta-analysis of treatments for child sexual abuse (Corcoran & Pillai, 2008), showing a mildly positive effect of parent-involved approaches at post-treatment, but not at follow-up. When only including treatment studies with a waitlist control group in their meta-analysis, Morina et al. (2016) did not find caregiver involvement to be a significant outcome moderator. Another meta-analysis of treatments for sexually abused children on PTSD symptoms, externalizing and internalizing problems even found a lower treatment effectiveness for interventions involving caregivers compared to interventions without caregiver involvement (Trask et al., 2011). However, the authors consider that these differences might result from the partly confounded type of control group in the studies. While most of the child-only treatment studies recruited no-treatment control groups, most of the caregiver-involving treatment studies utilized attention-placebo control groups.

With regard to depressive and anxiety disorders, the involvement of parents in treatment is also recommended and usual practice, although research is inconsistent. A meta-analysis on anxiety disorders found no benefit for parental involvement (Thulin et al., 2014). Dippel et al. (2022) identified a significant but small effect of family/caregivers' involvement for depressive disorders, though studies were highly heterogeneous regarding outcome measures as well as the extent of caregiver involvement in the treatment of depressed children and adolescents. It should be noted that the samples in both meta-analyses were not exclusively trauma-exposed children, but children/adolescents with a main diagnosis of anxiety or depression. Additional reasons for these inconsistent results may be that the aforementioned meta-analyses included studies with children and adolescents of different ages, different symptoms and various symptom severity following different types of traumatic events. Furthermore, they investigated treatments entailing various forms of caregiver involvement (e.g. caregivers attending sessions without children, sessions coinciding with children’s sessions, or sessions spent together with their children), different types of caregivers (e.g. biological parents, foster parents, youth welfare caregivers), caregivers with and without a psychopathology and different extents and qualities of caregiver involvement. The previous meta-analyses did not investigate moderators of the moderating effect of caregiver involvement. This is crucial, since interventions entailing caregiver involvement of any format and extent might not be effective in children of all ages with all sorts of mental disorders of varying severity after a myriad of traumatic events. Instead, research on evidence-based mental healthcare should thrive to answer “what treatment, by whom, is most effective for this individual with that specific problem and under which set of circumstances” (Paul, 1967, p. 111).

We conclude that potential effectiveness-determinants should be studied first before investigating the superiority of caregiver-involving interventions. Therefore, we aim to examine the effectiveness of interventions with caregiver involvement in more detail, by exploring the potential influence of children’s sex, age, symptom severity, the type of traumatic event, type of intervention, type of caregiver, format or extent of caregiver involvement. To our knowledge, the present systematic review and meta-analysis is the first to investigate potential moderators of the effectiveness of different kinds of caregiver-involving interventions for children and adolescents with different symptoms of mental disorders after traumatic events. Our exploratory approach could inform further investigations and might inform personalized mental health care by enabling individualized recommendations about caregiver involvement (Cuijpers et al., 2022).

## Methods

### Protocol and Registration

The present review was registered and approved by PROSPERO. More details are found under https://www.crd.york.ac.uk/prospero/display_record.php?ID=CRD42019129359.

### Eligibility Criteria

We set the eligibility criteria for studies following the Population, Intervention, Comparison, Outcome, and Study design (PICOS) scheme, as recommended by the Preferred Reporting Items for Systematic Reviews and Meta-analyses (PRISMA) group (Moher et al., 2009), including those studies whose participants were children or adolescents up to the age of 21 years who had been diagnosed with an adjustment disorder, (complex) PTSD, or another mental disorder caused by a traumatic experience. Diagnoses had to have relied on the Diagnostic and Statistical Manual of Mental Disorders (DSM) (American Psychiatric Association [APA], 2000) or the International Statistical Classification of Diseases and Related Health Problems (ICD) (World Health Organization [WHO], 2004) criteria and symptoms had to have been operationalized with evaluated clinical interviews, self-rating instruments or external rated assessment instruments (P). Children and adolescents had to have undergone a psychotherapeutic or psychological intervention entailing caregiver involvement in any format and to any extent (I). The intervention had to have been compared to a control condition with or without caregiver involvement (C). Studies had to have investigated evaluated outcome measures that operationalized the intervention’s efficacy as participants’ diagnostic status, symptom severity, or functioning level. Studies should have at least reported a baseline assessment and a post-measurement after the intervention (O). Randomized controlled trials (RCT), quasi-randomized controlled trials and efficacy studies were included. Single case, case control and cohort studies were excluded (S). Studies had to be in English or German. Dissertations were excluded. No other restrictions were applied.

These strict eligibility criteria resulted in only three eligible studies because in few studies all participating children fulfilled the diagnostic criteria of mental disorders. We therefore expanded our inclusion criteria to include studies in which children or adolescents showed symptoms of adjustment disorders, (complex) posttraumatic stress disorders, or other mental disorders caused by a traumatic experience within the clinical range. Studies had to have defined inclusion criteria for participants in terms of the number or severity of symptoms, or the samples’ average symptom levels had to have exceeded the clinical cut-offs of evaluated clinical interviews, self-rating instruments, or externally rated assessment instruments. This extension of our inclusion criteria resulted in *k* = 34 additional studies that could be included.

### Search

Journal articles starting from inception to July 19^th^ 2021 were searched in the databases PubMed, PsycINFO, ERIC, COCHRANE and PSYNDEX. Current reviews were screened for additional references. The search term is shown in Table S1 in the Supplemental material 1.

### Study Selection

Data selection followed the PRISMA guidelines (Moher et al., 2009). After searching databases, titles and abstracts of records were screened. Records had to (1) include children or adolescents up to the age of 21 years, (2) address children and adolescents’ mental health following traumatic experiences, (3) evaluate psychological interventions, and (4) be published in English or German to be included in the full-text screening. Thereafter, two independent researchers carried out a full-text screening based on the eligibility criteria. In case of discrepancies and disagreements about a study’s eligibility, they were discussed and resolved with the help of a senior member of the research team.

### Data Collection Process

Data were manually extracted from journal articles. Both study-level and effect size-level data were coded using standardized spreadsheets. When necessary data were unavailable, we contacted the corresponding authors. In case of no replies and missing data, the studies were not included in the respective analyses.

### Data Items

The extracted information included: post-intervention/follow-up intervention means or mean change scores, sample sizes, standard deviations or standard errors, publication year, study methodology, sample demographics, type of trauma, amount of participants fulfilling a diagnosis, outcome measures for child symptoms, type of caregiver involved, caregiver psychopathology, and extent of caregiver involvement. Separate effect sizes were computed for all available child-symptom outcomes and for child, parent and teacher reports. Pooled effect sizes were calculated if at least three effect sizes were available. In case both child and parent reports were available for one study, but not enough studies to calculate pooled effect sizes separately for child and parent reports, child reports were preferred since parent reports alone may underestimate symptomatology (Scheeringa et al., 2006).

### Risk of Bias

To assess the Risk of Bias (RoB) in each study, we used the Cochrane collaboration’s tool (Higgins et al., 2011) evaluating seven domains: Selection bias due to inadequate generation of a randomized sequence or due to inadequate concealment of allocations prior to assignment; performance bias due to prior knowledge of the allocated interventions by participants and personnel; detection bias due to knowledge of the allocated interventions by outcome assessors; attrition bias due to amount, nature or handling of incomplete outcome data; reporting bias due to selective outcome reporting; and bias due to other problems. Each domain was classified as having a low, high or unclear RoB. Two independent researchers did those assessments. Any disagreements were discussed and resolved through the assistance of a third team member.

### Synthesis of Results

Analyses were performed in R version 4.0.3 using the ‘meta’ (Schwarzer et al., 2015), ‘metafor’ (Viechtbauer, 2010) and ‘dmetar’ (Harrer et al., 2019) packages. In light of the small sample publications, small-sample bias correction has been applied by using bias corrected standardized mean differences (Hedges’ *g*) as effect size measures. If available, these were computed using (adjusted) post-intervention/follow-up intervention means, sample sizes and standard deviations or using mean change scores, sample sizes and standard errors. As recommended when there is heterogeneity and a low number of studies, the Hartung-Knapp-Sidik-Jonkman (HKSJ) method was used to estimate τ^2^ for random effects models (IntHout et al., 2014) with inverse-variance weighting. Heterogeneity between studies was assessed using the *I*^2^-statistics. The magnitude of heterogeneity caused by true variability was assessed with the *I*^2^ measure by Higgins et al. (2003), with *I*^*2*^ = 25% indicating low, 50% moderate and 75% substantial inconsistency. Studies were defined as outliers when their effect sizes’ 95% CI lay outside the 95% CI of the pooled effect. In case of outliers, all analyses were performed with and without outliers and diverging results were reported. To detect any small sample publication bias, funnel plots were generated and tested for asymmetry using Egger’s test of the intercept (Egger et al., 1997).

### Sensitivity Analyses

Sensitivity analyses were conducted to assess the robustness of all results. At first, the Maximum-Likelihood (ML) method was used to estimate τ^2^ in addition to the HKSJ (Wiksten et al., 2016) and results were compared. Moreover, results of studies with and without successfully conducted randomization, intent-to-treat (ITT) analysis, and with low, medium or high overall RoB were compared using the Q-test statistic for random effects models. Results were only reported if differences were detected.

### Subgroup Analyses

Random effects meta-regression analyses were performed to assess the association between effect sizes and the samples’ sex distribution and mean age. Samples’ symptom severity (grouped as all, above 50% or under 50% of participants fulfilling diagnostic criteria due to varying specifications by the original studies), the type of traumatic event (sexual abuse, domestic violence, other trauma types or mixed), type of intervention (trauma-focused or not) and the type of caregiver (parents, mothers or mixed/other caregivers) were included as categorical predictors of effect size. When studies involved both mothers and fathers, they were categorized as “parents” instead of “mothers”, even if predominantly mothers participated. Subgroup analyses were conducted to assess differences in subsamples regarding the following variables: Type of control condition (active, i.e. other interventions, passive, e.g. supportive contact or usual care, or waitlist), format of caregiver involvement (parallel sessions, conjoint sessions or both) and extent of caregiver involvement (100%, indicating that caregivers attended all sessions or as many sessions as children and adolescents did, or less than 100%, this grouping was due to varying specifications by the original studies too). Analyses regarding caregiver psychopathology and quality of involvement could not be investigated due to insufficient information provided by the studies. Only significant results of meta-regression and subgroup analyses were reported below. Results of all sensitivity and subgroup analyses can be found in Supplemental material 1, Tables S4 and S5.

## Results

### Study Selection

**Fig. 1:**
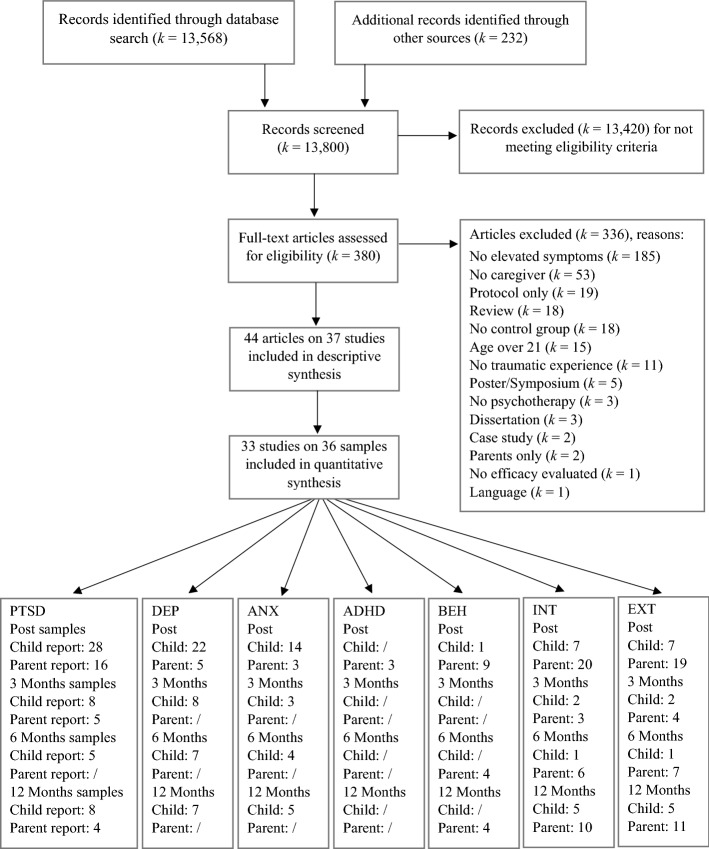
PRISMA flow chart

A total of 13,800 references were retrieved in databases and additional sources. After title and abstract screening, 13,420 references were excluded. From the remaining 380 publications within the full-text screening, 336 were excluded (see reasons in PRISMA flow diagram in Fig. 1). A total of 44 articles on *k* = 37 studies fulfilled our extended eligibility criteria (see methods chapter ‘eligibility criteria’) and were retained for descriptive analysis. These studies and their references are listed in Supplemental material 2. *K* = 33 studies provided sufficient data for quantitative analysis. One article (Dorsey et al., 2020) reported on four independent subsamples that were included separately in our quantitative analysis, resulting in 36 samples (a: Kenya-Urban, b: Kenya-Rural, c: Tanzania-Urban, d: Tanzania-Rural).

### Study Characteristics

The 44 included articles were published between 1996 and 2021. A total of *N* = 3845 children or adolescents participated in the studies, with a mean of *N* = 103.92 (range 24 to 640) per study. Age of included participants ranged from 1 to 18 years. Sample’s mean ages were reported for *k* = 33 studies, ranging from *M* = 3.30 to *M* = 16.02 years with an overall mean of 10.14 years (*SD* = 3.21). Sex distributions were reported for *k* = 36 studies with an overall mean of 56.75% (*SD* = 17.85) female participants, range 27.70% to 100.00%. Most studies included participants exposed to mixed traumatic events (45.5%), followed by sexual abuse (24.2%), domestic violence (12.1%) and physical abuse, community violence, hurricanes, explosions, accidents and parental loss (3.0% respectively).

The *k* = 37 studies investigated 19 different interventions with trauma focused CBT interventions being the most frequently investigated treatments (*k* = 17) including 14 studies investigating Tf-CBT by Cohen et al. (2016). This was followed by Cognitive Behavioral Therapy (CBT) interventions (*k* = 4), abuse-specific CBT interventions (*k* = 3), Bounce Back intervention (*k* = 2) and Risk Reduction through Family Therapy (*k* = 2). Active treatments served as control conditions in *k* = 15 studies, passive interventions in *k* = 13 and waitlist condition was investigated in *k* = 9 studies. The most frequently investigated active control interventions were Eye Movement Desensitization and Reprocessing (EMDR) (*k* = 3), Child CBT (*k* = 2), Child centered therapy (*k* = 2) and Tf-CBT by Cohen et al. (2016) (*k* = 2). Regarding the type of caregiver involved (i.e. biological, foster), *k* = 32 studies reported some information. Different types of caregivers are reported in *k* = 15 studies, parents served as caregivers in *k* = 10 and mothers in *k* = 7 studies. Only *k* = 5 studies reported on caregivers’ baseline psychopathology, *k* = 8 on the quality or adherence of caregiver involvement. Additional information is found in Table S2 (Supplemental material 1).

### Risk of Bias

**Fig. 2:**
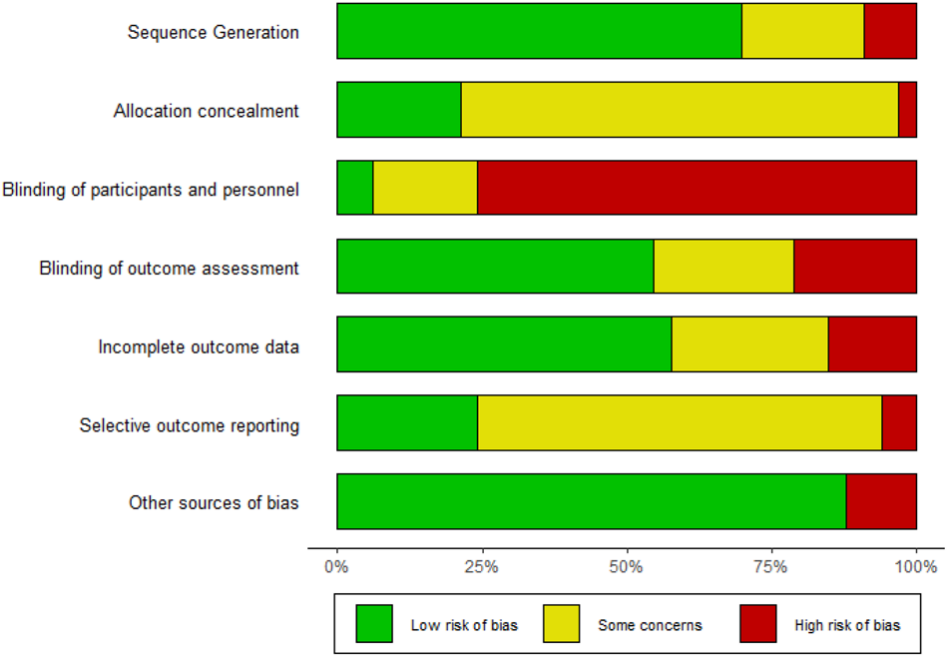
Summary of the Risk of Bias Assessment of studies included in quantitative analysis

Figure 2 summarizes the RoB of all studies included in quantitative analysis. Only two studies had a low RoB due to blinding of participants and personnel. It should be noted that especially blinding of personnel is rarely possible in psychotherapy research studies. Only few studies reported on a study protocol. Therefore, RoB due to selective outcome reporting was difficult to assess for those studies. In Table S3 (Supplemental material 1), RoB assessments for all *k* = 37 studies are shown with reasons. Overall, *k* = 23 had a high, *k* = 4 a medium and *k* = 10 a low RoB.

### Results of Individual Studies and Meta-Analytical Synthesis

**Fig. 3:**
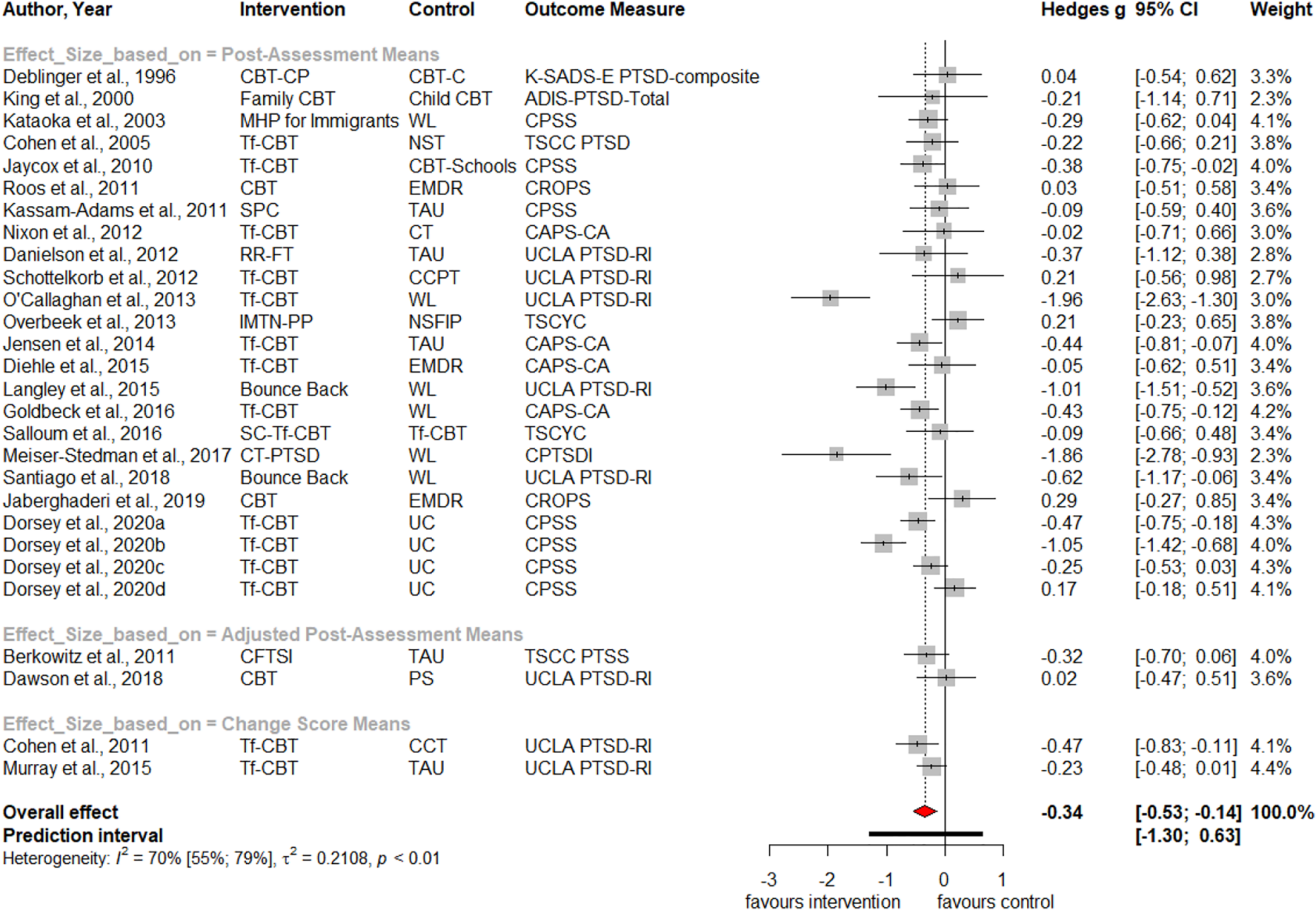
Forest Plot for child-reported PTSD symptoms at post-treatment

**Fig. 4:**
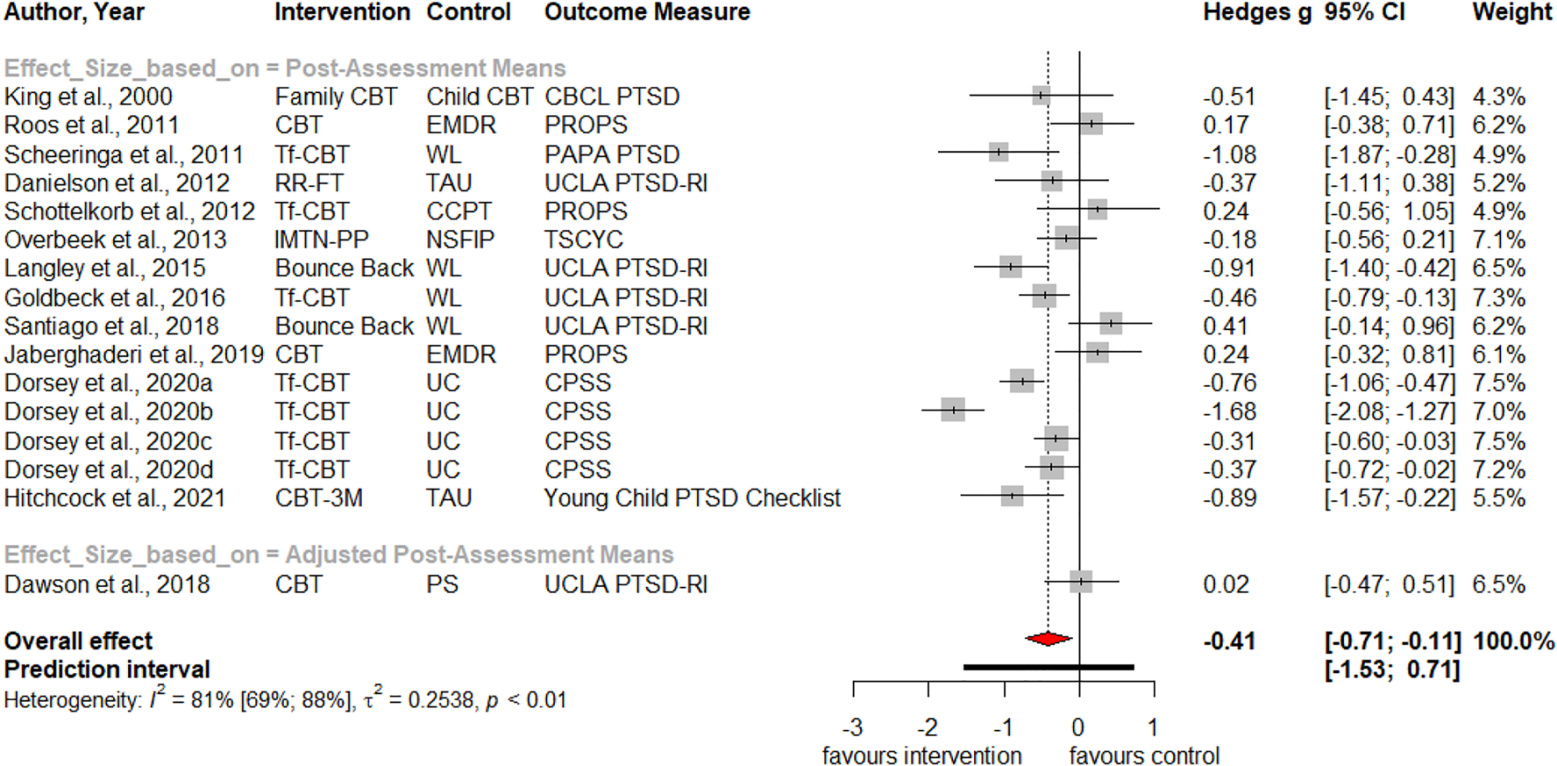
Forest Plot for parent-reported PTSD symptoms at post-treatment

**Fig. 5:**
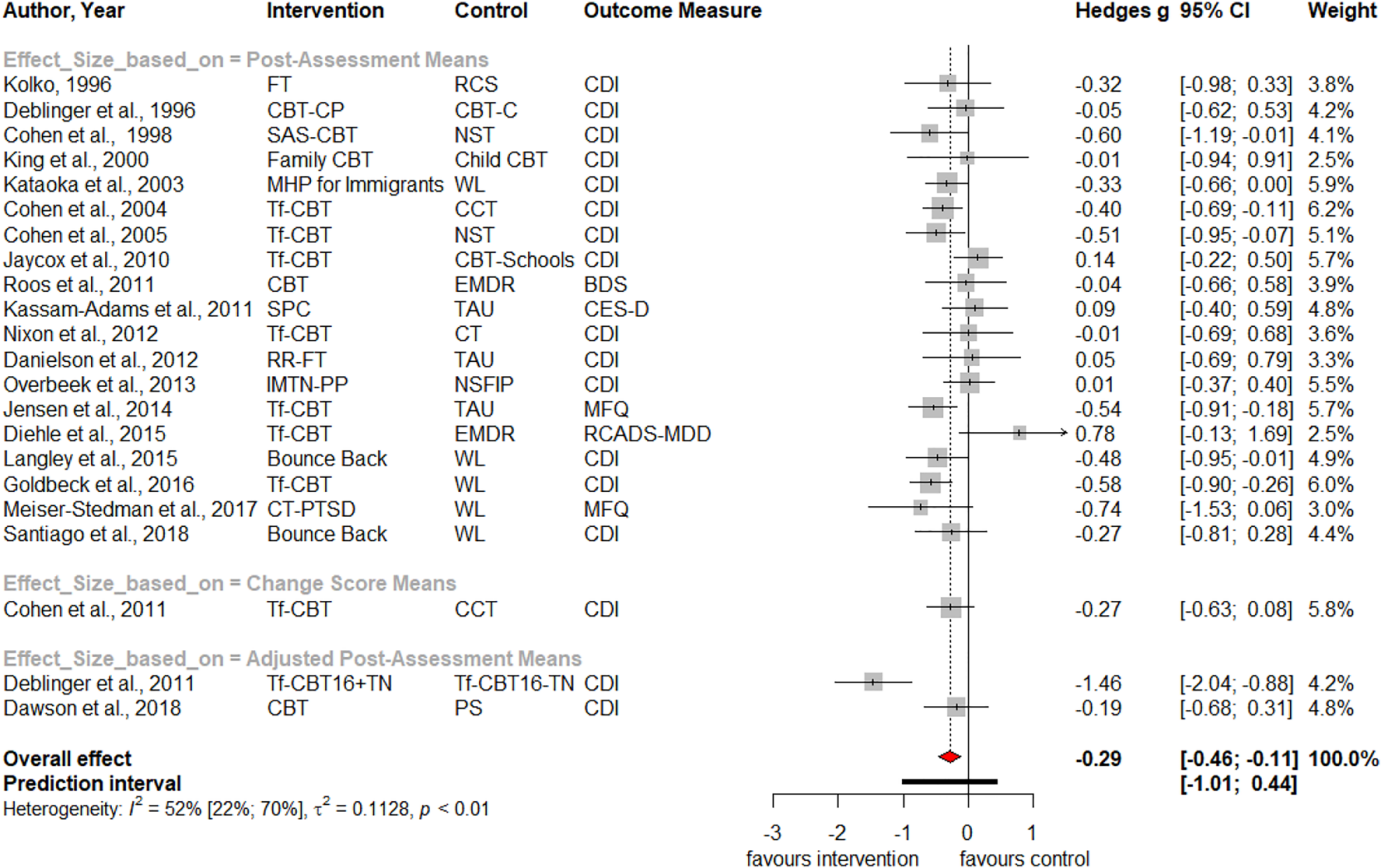
Forest Plot for child-reported depressive symptoms at post-treatment

**Fig. 6:**
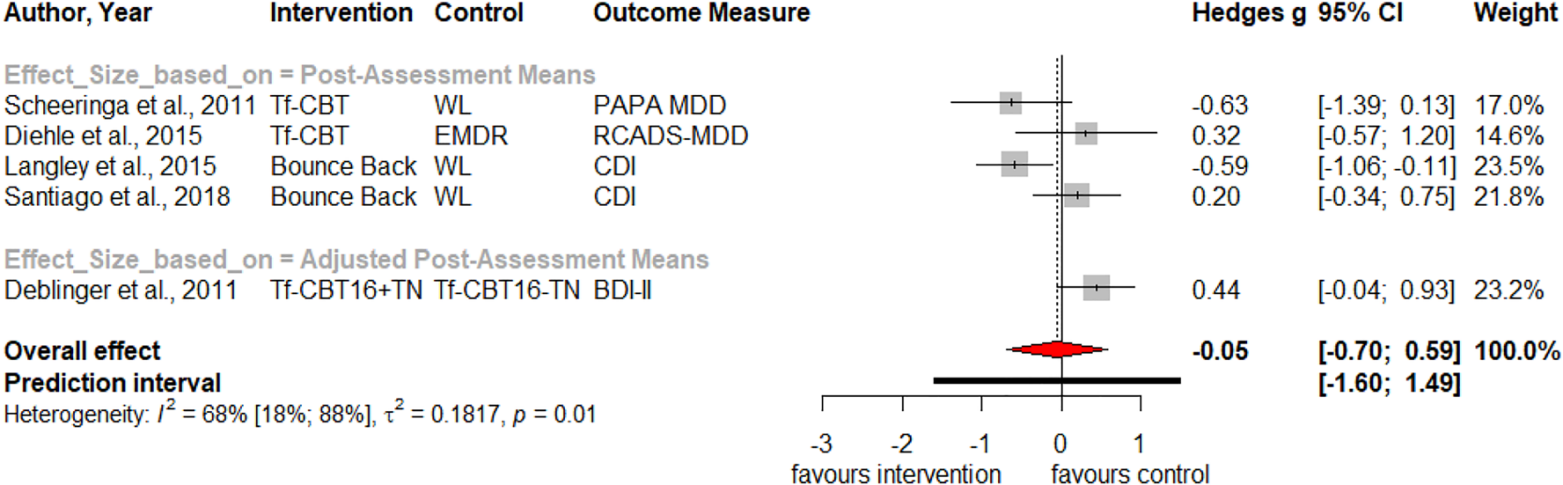
Forest Plot for parent-reported depressive symptoms at post-treatment

**Fig. 7:**
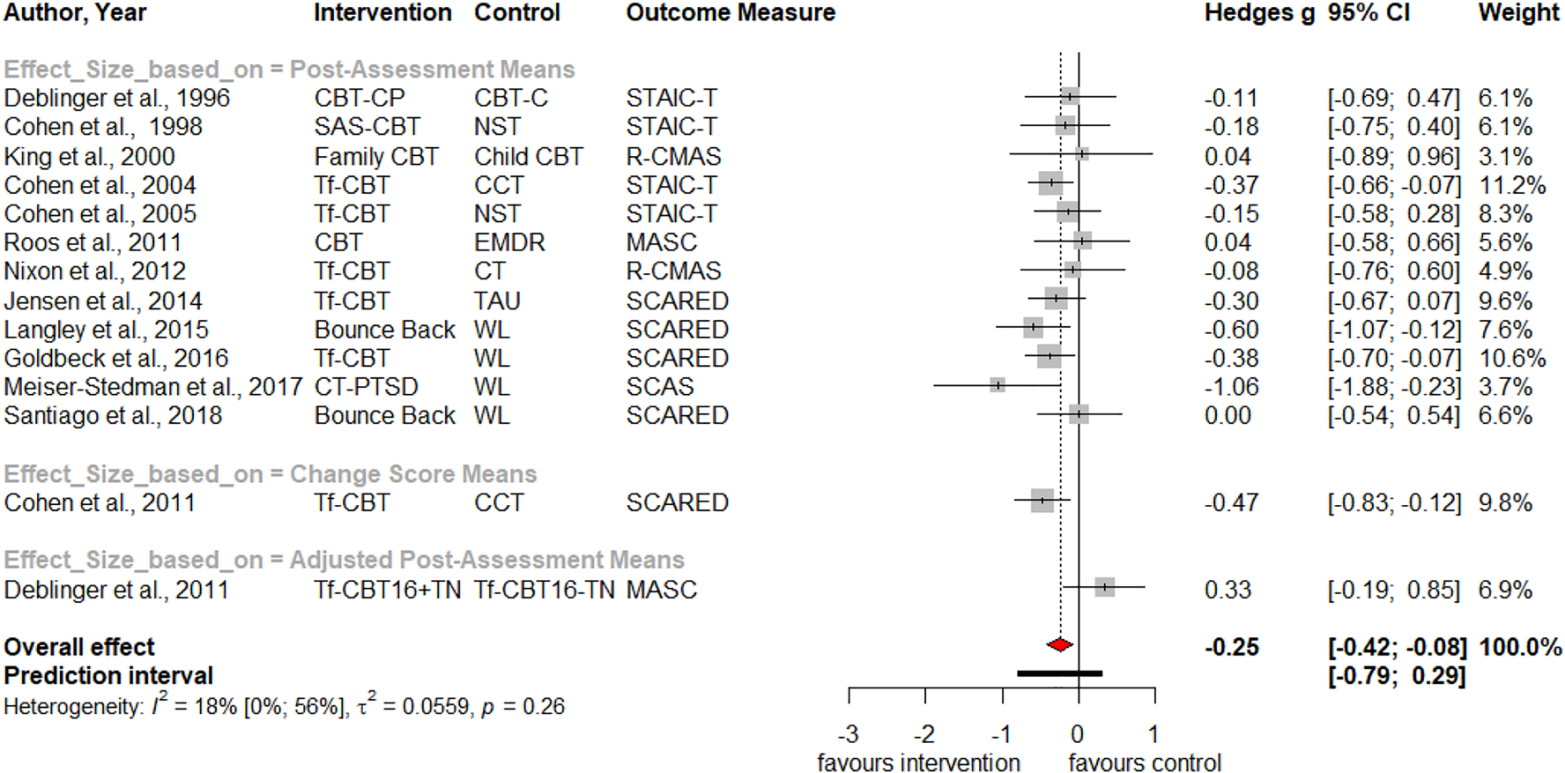
Forest Plot for child-reported anxiety symptoms at post-treatment

**Fig. 8:**
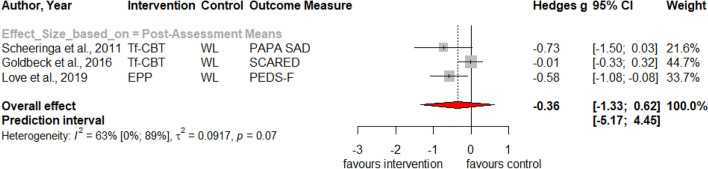
Forest Plot for parent-reported anxiety symptoms at post-treatment

**Fig. 9:**
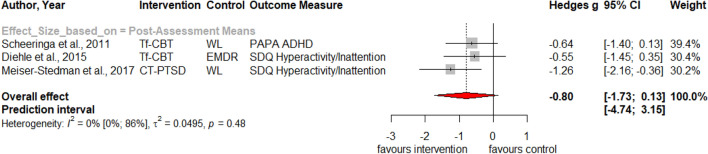
Forest Plot for parent-reported ADHD symptoms at post-treatment

**Fig. 10:**
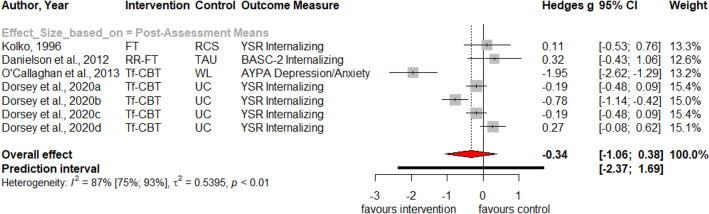
Forest Plot for child-reported internalizing symptoms at post-treatment

**Fig. 11:**
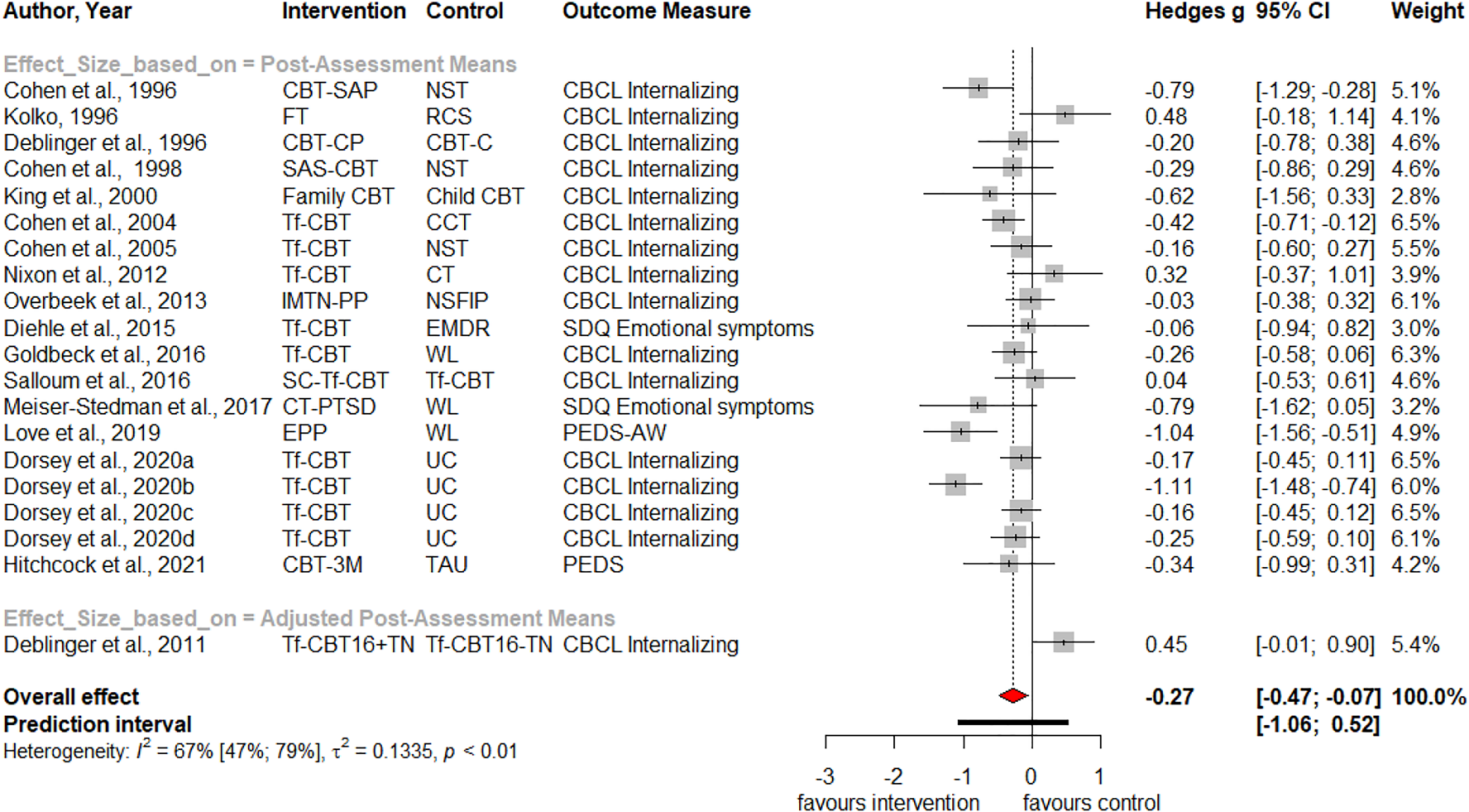
Forest Plot for parent-reported internalizing symptoms at post-treatment

**Fig. 12:**
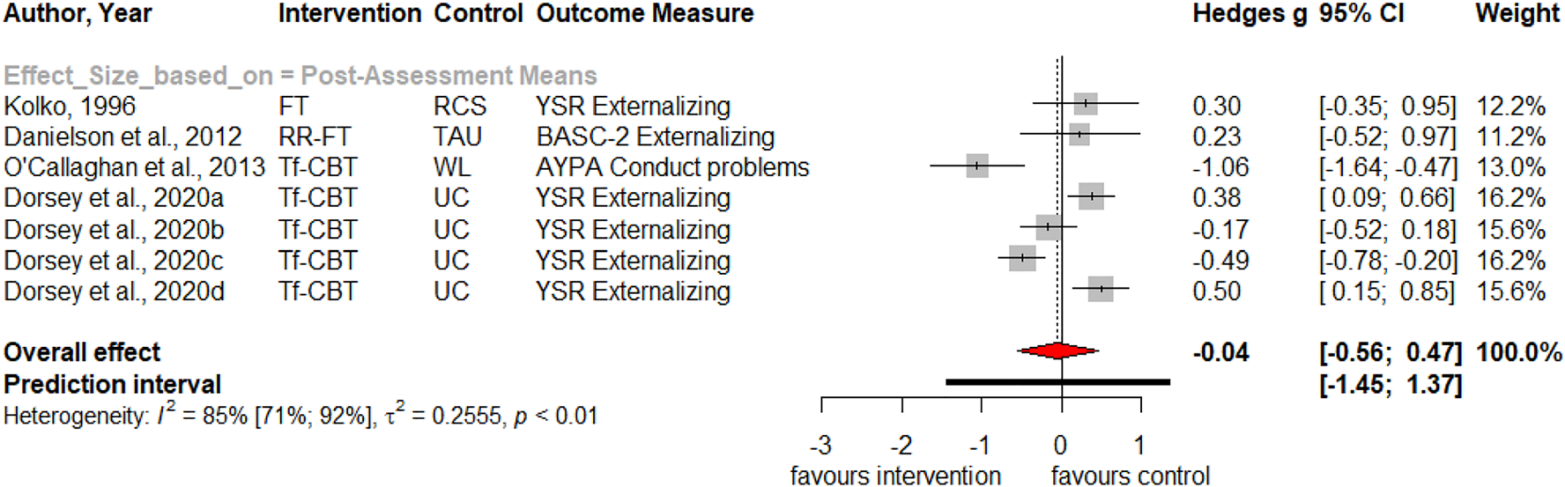
Forest Plot for child-reported externalizing symptoms at post-treatment

**Fig. 13:**
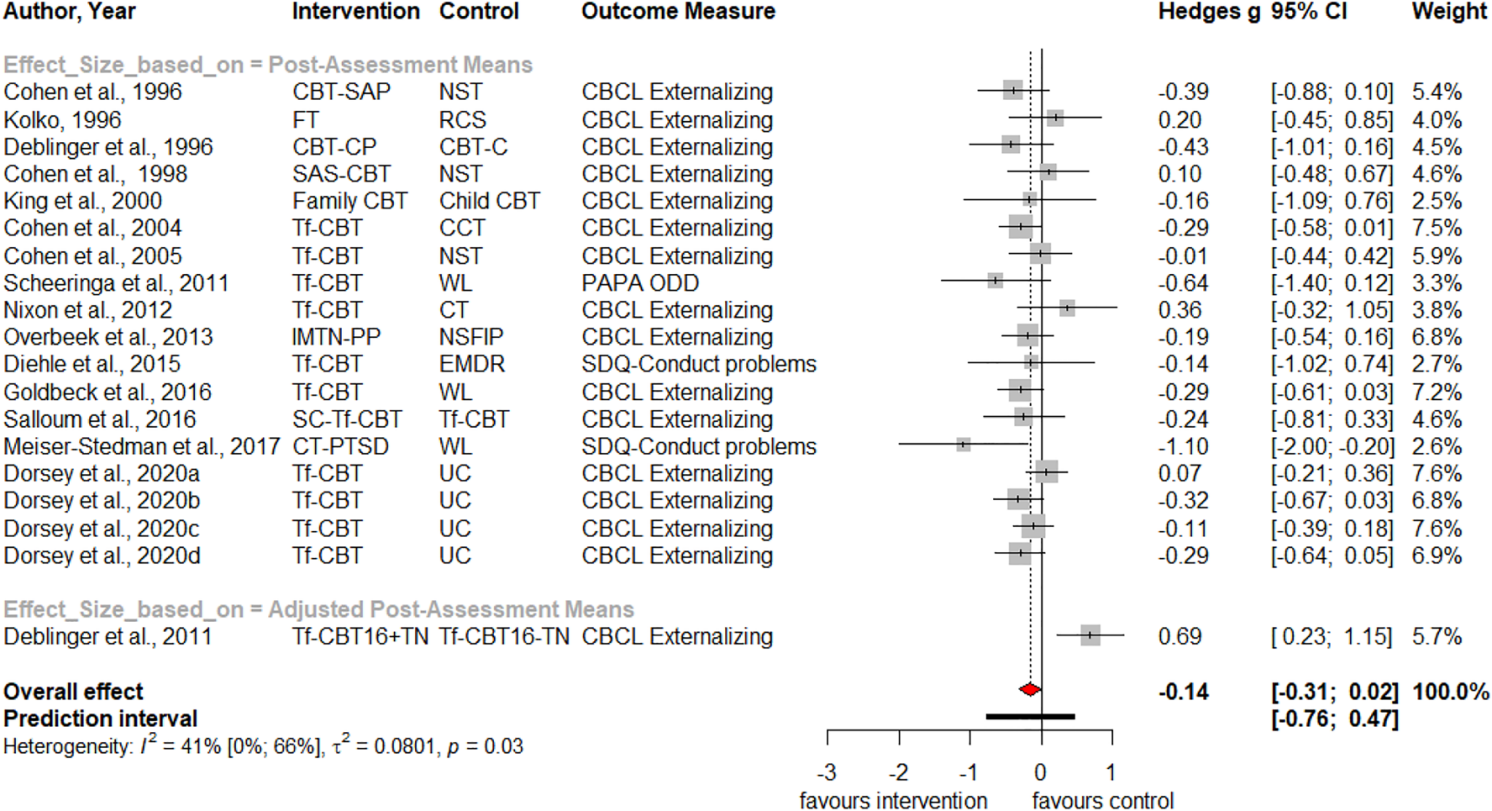
Forest Plot for parent-reported externalizing symptoms at post-treatment

**Fig. 14:**
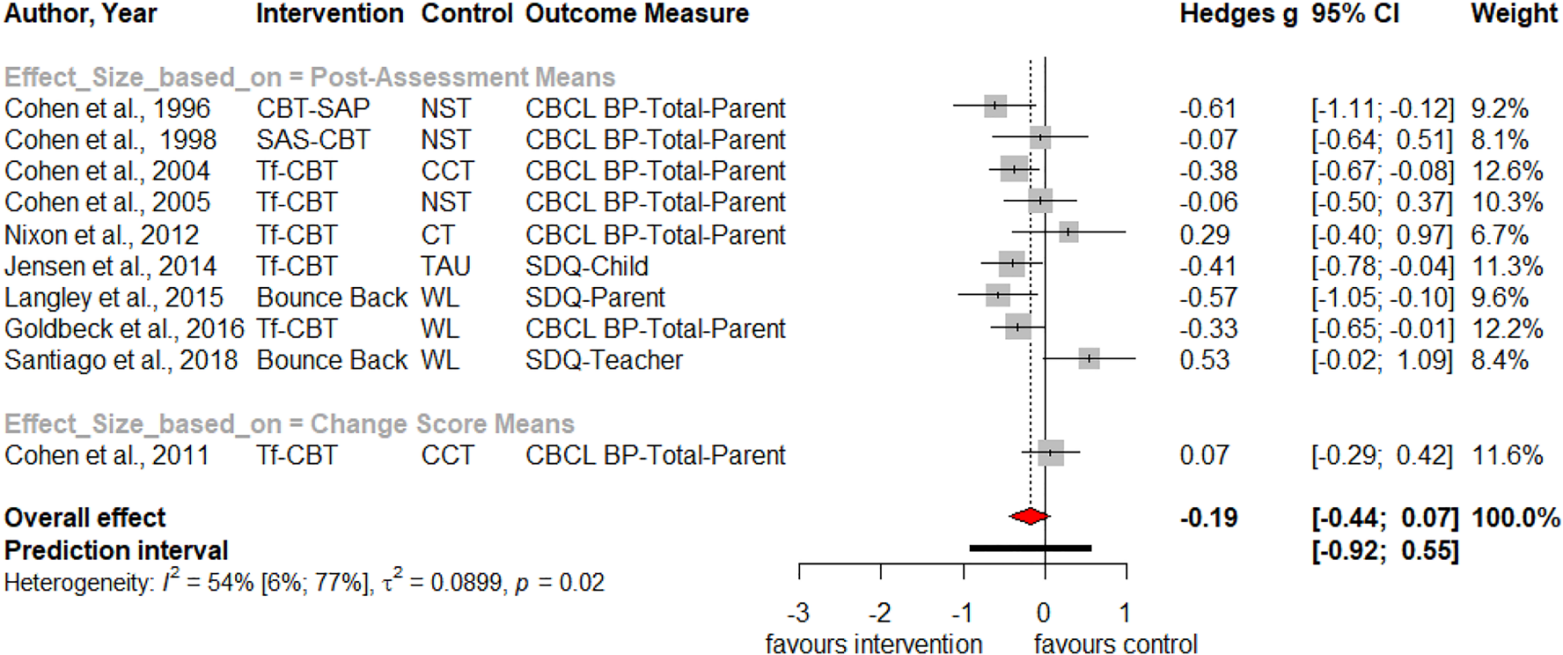
Forest Plot for behavior problems at post-treatment

Table 1 summarizes the results for symptoms of PTSD, depression, anxiety, attention deficit hyperactivity disorder (ADHD), internalizing, externalizing symptoms and behavior problems at post-treatment and at three, six and twelve months follow-up. Forest plots for post-assessment analyses are found in Figs. 3, 4, 5, 6, 7, 8, 9, 10, 11, 12, 13,14. Forest plots for follow-up assessment analyses are shown in Figures S1-S21 in the Supplemental material 3. The pooled effect size was significant and robust at post-treatment for child-reported PTSD, *g* = − 0.34 (95% *CI* = − 0.53; − 0.14), parent-reported PTSD, *g* = − 0.41 (95% *CI* = − 0.71; − 0.11), child-reported depression, *g* =  −  0.29 (95% *CI* = − 0.46; − 0.11), child-reported anxiety, *g* = − 0.25 (95% *CI* = − 0.42; − 0.08), and parent-reported internalizing symptoms, *g* = − 0.27 (95% *CI* = − 0.47; − 0.07). The only significant effect size at follow-up was found for child-reported PTSD symptoms 12 months post-treatment, *g* = − 0.37 (95% *CI* = − 0.67; − 0.07). Negative effect sizes indicate greater effectiveness for interventions with caregiver involvement compared to control conditions.

**Table 1:** Results of all post- and follow-up meta-analyses for PTSD, depression, anxiety, ADHD, internalizing, externalizing and behavior problems

	PTSD		DEP		ANX			ADHD	INT		EXT		BEH
	Child	Parent	Child	Parent	Child	Parent		Parent	Child	Parent	Child	Parent	Child
Post-assessment	***k* = 28** ***g* = -0.34** ***p* = .002** *I*^2 ^= 69.5% *Q* = 88.65 *p* < .001	***k* = 16** ***g* = -0.41** ***p* = .011** *I*^2 ^= 80.5% *Q* = 76.99 *p* < .001	***k* = 22** ***g* = -0.29** ***p* = .003** *I*^2 ^= 51.9% *Q* = 43.66 *p* = .003	*k* = 5 *g* = -0.05 *p* = .833 *I*^2^ = 68.2% *Q* = 12.57 *p* = .014	***k* = 14** ***g* = -0.25** ***p* = .007** *I*^2 ^=18.0% *Q* = 15.86 *p* = .257	*k* = 3 *g* = -0.36 *p* = .256 *I*^2 ^=63.3% *Q* = 5.44 *p* = .066		*k* = 3 *g* = -0.80 *p* = .066 *I*^2 ^=0.0% *Q* = 1.48 *p* = .477	*k* = 7 *g* = -0.34 *p* = .293 *I*^2 ^= 86.9% *Q* = 45.86 *p* < .001	***k* = 20** ***g* = -0.27** ***p* = .011** *I*^2 ^=66.6% *Q* = 56.90 *p* < .001	*k* = 7 *g* = -0.04 *p* = .849 *I*^2 ^=85.1% *Q* = 40.34 *p* < .001	*k* = 19 *g* = -0.15 *p* = .077 *I*^2 ^=41.3% *Q* = 30.67 *p* = .031	*k* = 10 *g* = -0.19 *p* = .130 *I*^2 ^=54.0% *Q* = 19.57 *p* = .021
Without outliers	***k* = 25** ***g* = -0.23** ***p* < .001** *I*^2^ = 35.2% *Q* = 37.01 *p* = .044	***k* = 15** ***g* = -0.32** ***p* = .016** *I*^2^ = 64.2% *Q* = 39.10 *p* < .001	***k* = 21** ***g* = -0.25** ***p* = .001** *I*^2^ = 28.8% *Q* = 28.08 *p* = .107						*k* = 6 *g* = -0.12 *p* = .495 *I*^2^ = 74.6% *Q* = 19.67 *p* = .001	***k* = 17** ***g* = -0.21** ***p* = .007** *I*^2^ = 13.2% *Q* = 18.43 *p* = .299		***k* = 18** ***g* = -0.19** ***p* = .006** *I*^2^ = 2.1% *Q* = 17.37 *p* = .439	
3 months folllow-up	*k* = 8 *g* = -0.12 *p* = .188	*k* = 5 *g* = -0.07 *p* = .689	*k* = 8 *g* = 0.01 *p* = .970		*k* = 3 *g* = -0.27 *p* = .117				*k* = 5 *g* = -0.13 *p* = .249		*k* = 6 *g* = -0.08 *p* = .497		
6 months follow-up	*k* = 5 *g* = -0.06 *p* = .674		*k* = 7 *g* = -0.02 *p* = .874		*k* = 4 *g* = 0.00 *p* = .996				*k* = 7 *g* = 0.04 *p* = .667		*k* = 8 *g* = 0.04 *p* = .568		*k* = 4 *g* = -0.05 *p* = .654
12 months follow-up	***k* = 8** ***g* = -0.37** ***p* = .023**	*k* = 4 *g* = -0.66 *p* = .070	*k* = 7 *g* = -0.13 *p* = .262		*k* = 5 *g* = -0.14 *p* = .091				*k* = 5 *g* = -0.35 *p* = .114	*k* = 10 *g* = -0.20 *p* = .132	*k* = 5 *g* = -0.15 *p* = .381	*k* = 11 *g* = -0.17 *p* = .066	*k* = 4 *g* = -0.08 *p* = .304

Statistical significant results are highlighted in bold font

Significant heterogeneity is found for child- and parent-reported PTSD, depression, internalizing, externalizing symptoms and behavior problems. Analyses without outliers reduces heterogeneity, resulting in insignificant heterogeneity for child-reported depression, parent-reported internalizing and externalizing symptoms. Visual inspections of the funnel plots (Figures S22-S33 in Supplemental material 4) and non-significant Egger’s tests of the intercept did not indicate the presence of funnel plot asymmetries.

### Sensitivity Analyses

For ADHD symptoms and behavior problems, CI of the pooled effect sizes differed substantially when the ML method was used (*CI* = − 1.28; − 0.30) to estimate τ^2^ compared to the HKSJ method (*CI* = −1.73; 0.13). For parent-reported PTSD symptoms, we noted a significant difference between studies with low (*k* = 6); *g* = − 0.73; 95%-CI = − 1.27; − 0.19; medium (*k* = 1); *g* = 0.17; 95%-*CI* = − 0.38; 0.71; and high RoB (*k* = 9); *g* = − 0.21; 95%-*CI* = − 0.61; 0.19; *Q* = 7.27, *df* = 2, *p* = .026. This was also the case for parent-reported anxiety symptoms with differences between studies with low (*k* = 1); *g* = − 0.01; 95%-*CI* = − 0.33; 0.32; and high RoB (*k* = 2); g = − 0.63; 95%-CI = − 1.51; 0.25; *Q* = 12.26, *df* = 1, *p* < .001; as well as for behavior problems with differences between studies with high (*k* = 6); *g* = − 0.21; 95%-*CI* = − 0.65; 0.22; medium (*k* = 2); *g* = 0.11; 95%-*CI* = − 1.04; 1.27; and low RoB (*k* = 2); *g* = − 0.36; 95%-*CI* = − 0.86; 0.13; *Q* = 23.43, *df* = 2, *p* < .001. For externalizing symptoms, the pooled effect size differed between studies with (*k* = 6); *g* = 0.09; 95%-*CI* = − 0.32; 0.52; and without (*k* = 1) ITT analysis; *g* = − 1.06; 95%-*CI* = − 1.64; – 0.47; *Q* = 11.54, *df* = 1, *p* < .001.

### Subgroup Analyses

For child-reported PTSD symptoms, the involvement of over 50% participants who fulfilled the diagnostic criteria for mental disorders; ß = 1.527; *t* = 4.48; *p* = .001; and the involvement of under 50%; ß = 1.743; *t* = 5.09; *p* < .001, *F*(2, 10) = 13.34; *p* = .001; were significant predictors of effect size when the outliers were included in the analysis. This was also the case for child-reported depressive symptoms: The involvement of under 50% participants who fulfilled the diagnostic criteria for mental disorders was a significant predictor of effect size (*k* = 4); ß = 0.902; *t* = 2.49; *p* = .035. This moderation of effect size was significant; *F*(2, 9) = 6.55; *p* = .018. For child-reported symptoms of anxiety disorders, the involvement of over 50% participants who fulfilled the diagnostic criteria for mental disorders was a significant predictor of effect size (*k* = 6); ß = 0.736; *t* = 2.85; *p* = .029; as was the involvement of under 50% (*k* = 1); ß = 1.096; *t* = 3.42; *p* = .014. This moderation of effect size was significant; *F*(2, 6) = 5.91; *p* = .038. For caregiver-involving interventions, this indicates higher benefits for children that fulfill diagnostic criteria compared to children who do not fulfill full criteria.

When the outliers were included in the analysis, the sex distribution in the included studies; ß = − 0.014; *F*(1, 26) = 5.94; *p* = .022; was a significant predictor of the effect size for child-reported PTSD symptoms. For parent-reported internalizing, the sex distribution in the included studies was a significant predictor effect size (*k* = 16); ß = − 0.009; *F*(1, 14) = 4.83; *p* = .045. For caregiver-involving interventions, this indicates higher benefits for female children compared to children with other sex. For child-reported symptoms of depressive disorders, the involvement of mothers (*k* = 3); ß = 0.479; *t* = 2.29; *p* = .038; and the involvement of parents (*k* = 7); ß = 0.475; *t* = 2.76; *p* = .015; were significant predictors of effect size; *F*(2, 14) = 4.63; *p* = .029.

For parent-reported anxiety symptoms, studies reporting on conjoint sessions for caregivers and children (*k* = 2); *g* = − 0.63; 95%-*CI* = − 1.51; 0.25; differed from the one study with both parallel and conjoint sessions (*k* = 1); *g* = − 0.01; 95%-*CI* = − 0.33; 0.32; *Q* = 12.26, *df* = 1, *p* < .001. This was also the case for externalizing symptoms: The one study reporting on conjoint sessions for caregivers and children (*k* = 1); *g* = 0.30; 95%-*CI* = − 0.35; 0.95; differed from the study with parallel sessions (*k* = 1); *g* = − 1.06; 95%-*CI* = − 1.64; – 0.47; and from the studies with both conjoint and parallel sessions (*k* = 5); *g* = 0.07; 95%-*CI* = – 0.46; 0.60; *Q* = 12.50, *df* = 2, *p* < .001.

Significant subgroup differences were found between studies with active (*k* = 10); *g* = − 0.12; 95%-*CI* = − 0.31; 0.06; passive (*k* = 12); *g* = − 0.26; 95%-*CI* = − 0.48; – 0.05; and waitlist control conditions (*k* = 6); *g* = − 0.96; 95%-*CI* = − 1.70; – 0.22; *Q* = 8.04, *df* = 2, *p* = .002; for child-reported PTSD symptoms. This was also the case for parent-reported PTSD symptoms: Effect sizes of studies with active (*k* = 4); *g* = 0.10; 95%-*CI* = − 0.37; 0.58; passive (*k* = 8); *g* = – 0.57; 95%-*CI* = − 1.02; – 0.11; and waitlist control conditions (*k* = 4); *g* = − 0.49; 95%-*CI* = − 1.52; 0.55; differed substantially; *Q* = 8.57, *df* = 2, *p* = .014. In the analysis of child-reported depressive symptoms without the outlier study, we noted a significant subgroup difference between studies with active (*k* = 8); *g* = − 0.07; 95%-*CI* = − 0.33; 0.18; passive (*k* = 8); *g* = − 0.26; 95%-*CI* = − 0.50; − 0.03; and waitlist control conditions (*k* = 5); *g* = − 0.46; 95%-*CI* = − 0.65; – 0.26; *Q* = 9.25, *df* = 2, *p* = .010. Lastly, we noted a significant subgroup difference between studies with passive (*k* = 6); *g* = 0.09; 95%-*CI* = − 0.32; 0.52; and waitlist control conditions (*k* = 1); *g* = − 1.05; 95%-*CI* = − 1.64; – 0.47; *Q* = 11.54, *df* = 1, *p* < .001; investigating externalizing symptoms.

For parent-reported depressive symptoms, we noted a significant subgroup difference between studies with waitlist control conditions and conjoint sessions for caregivers and children (*k* = 3); *g* = − 0.32; 95%-*CI* = − 1.49; 0.85; and studies with active control conditions and both parallel and conjoint sessions (*k* = 2); *g* = 0.41; 95%-*CI* = − 0.27; 1.10; *Q* = 6.98, *df* = 1, *p* = .008; not allowing conclusions on the actual differentiating aspect. This was also the case for child-reported internalizing symptoms and moreover, sensitivity and subgroup analyses with and without the outlier led to diverging results. The inclusion of an outlier, the study by O'Callaghan et al. (2013), the only one without ITT analysis and with a waitlist control condition, mixed trauma types and parallel sessions for children and caregivers, led to significant subgroup differences; *Q* = 23.45, *df* = 1, *p* < .001.

## Discussion

Results of meta-analyses examining the superiority of caregiver-involving interventions are inconsistent. Moreover, they do not answer which intervention entailing caregiver involvement of what format and extent is effective for children of which ages with which symptoms of mental disorders following what type of traumatic event. The present systematic review and meta-analysis therefore investigated the effectiveness of caregiver-involving interventions for trauma-affected children and adolescents and examined potential moderators influencing the effectiveness.

A total of 44 articles reporting on 37 studies investigating 19 different interventions involving caregivers were included, and 33 studies with 36 independent samples were analyzed. The majority of studies investigated Tf-CBT by Cohen et al. (2016), followed by CBT interventions or family therapy. Active control conditions included EMDR, child CBT and Tf-CBT without or with less caregiver involvement. Most studies involved different types of caregivers, followed by parents or mothers alone. A high proportion of the analyzed studies had a high RoB, but this was predominantly due to the lack of blinding of participants and personnel, a procedure that is rarely possible or successful in psychotherapy research studies. Sensitivity analyses indicated robust results.

Our meta-analyses suggest greater symptom reductions through interventions involving caregivers than through control conditions for child- and parent-reported PTSD symptoms, child-reported anxiety and depressive symptoms and parent-reported internalizing symptoms. This indicates that caregiver-involving interventions are indeed effective. However, the only significant effect at follow-up assessments was detected for child-reported PTSD symptoms at 12 months post-treatment. This finding is partly in line with results of previous meta-analyses that could not replicate a moderating effect of caregiver involvement on treatment effectiveness in their follow-up analyses (Gillies et al., 2016; Gutermann et al., 2017). Caregiver involvement might thus accelerate the symptom reduction, however, not be mandatory in the long term. Another explanation for the result could be that children are less reliant on parents' support for their recovery after treatment completion compared to during treatment. Future studies should investigate whether caregiver involvement is especially beneficial at specific treatment phases, for example the trauma processing sessions (Yasinski et al., 2016).

We considered potential determinants of the effectiveness of interventions with caregiver involvement. Sex differences were identified for child-reported PTSD symptoms and parent-reported internalizing symptoms, both indicating that females might benefit more from caregiver-involving interventions. It should be considered that a meta-analysis on trauma-focused interventions for adults with PTSD identified gender differences with larger symptom reductions for females (Wade et al., 2016). Thus, the moderating effect in our analyses might not be specific to caregiver involvement, but may exist for trauma interventions in general. However, the meta-analysis by Gutermann et al. (2016) on children and adolescents with PTSD found a moderating effect of sex in only one of four analyses. Another explanation might be that the involvement of caregivers in children’s and adolescents’ treatments increases the actual or perceived amount of social support. Several studies show that higher social support during PTSD treatment is associated with better treatment responses (Birkeland et al., 2020; Fletcher et al., 2021; Price et al., 2018). Moreover, results of a prospective cohort study indicate that females’ mental health benefits more from social support (Milner et al., 2016). As a result, females might show a higher benefit from the involvement of caregivers in their treatment compared to males. A recent study (Ascienzo et al., 2022) examined gender differences during isolated phases of Tf-CBT by Cohen et al. (2016). While both male and female children and adolescents showed substantial symptom reduction, results indicated that the processing and integration phase might be particularly beneficial for girls (Ascienzo et al., 2022). Therefore, future studies should further investigate sex differences in treatment response during specific treatment phases and consider a potential interaction with caregiver involvement. Control variables such as trauma type should be considered in this process.

The involvement of more participants fulfilling diagnostic criteria as an indicator of symptom severity resulted in higher effect sizes favouring interventions with caregivers for child reports on PTSD, depressive and anxiety symptoms. This is in line with the results of a meta-analysis (Gillies et al., 2016), showing larger symptom reductions in children diagnosed with PTSD compared to those exposed to trauma without PTSD. However, among those participants with no diagnosis, some might have and other not have symptom-related functional impairment. The inclusion of those not impaired might bias the estimation of effectiveness since they are unlikely to exhibit considerable symptom reductions following interventions. In future, dimensional assessments and consideration of symptom severity and functional impairment could enhance validity.

Regarding the format of caregiver involvement, heterogeneous results were found and should be considered with caution. The effect size for parent-reported depressive symptoms is greater for interventions with conjoint sessions for children and caregivers, while the effect size for child-reported internalizing symptoms is greater for interventions with parallel sessions. Future studies should seek to determine which format is best suited for which phase of therapy. In addition, it remains to be answered whether children and adolescents benefit more or less from the involvement of specific caregivers. In our analyses, studies that included only mothers and only parents showed smaller effect sizes than studies with various types of caregivers for child-reported depressive symptoms. Further criteria for inclusion (e.g., the extent of their dysfunctional cognitions and reactions related to their child's trauma) should be identified.

No differences or inconclusive findings were detected for participants’ age, the type of trauma they experienced, whether the interventions are trauma-focused or not, and the amount of caregiver involvement. We were unable to investigate caregivers’ psychopathology or the quality of their involvement as potential moderators since so few studies provided the required information. Future studies should report on these important aspects of treatment protocols and their fidelity. Thereby we could answer additional important questions like “Which parts of parent interventions/components are most important for which caregivers?” Several interacting moderators could as well be of interest. Results of these analyses might inform practitioners’ decisions regarding the involvement of caregivers in mental health care.

The present meta-analysis is limited by the missing information in the publications we included; four studies could not be included in our quantitative analysis, and some of the moderator analyses we had planned were not feasible (caregivers’ psychopathology, quality of caregiver involvement). This is probably the result of the fact that few of the included studies specifically investigated whether interventions with caregiver involvement are effective but compared the outcomes of interventions in general. Due to the exploratory nature and the small number of included studies, corrections for multiple comparisons were not performed. The results on potential moderators of effect size should therefore be considered with caution. No definite conclusions can be drawn about whether the differences are due to the extent to which caregivers were included in the treatment or due to the specific intervention or aspects of the study design. Most studies did not conduct blinding of participants and personnel. For this reason, interventions’ effect sizes might be biased due to higher expectancies. Significant heterogeneity is found and might be the result of differences in studies’ populations, interventions and their doses, measurement instruments or methodological issues, thus posing interpretive challenges. The search term focuses on PTSD as an outcome of interest. Explicitly entering search terms like ‘depression’ might have shown off additional relevant studies.

## Conclusion

All in all, treatments involving caregivers for children and adolescents suffering from serious symptoms of mental disorders after traumatic events seem to be effective. They might be particularly beneficial for female children and adolescents and for those who fulfill the diagnostic criteria of mental disorders. Despite these initial findings, the results of our systematic review and meta-analysis raise important questions which cannot be answered with the currently available data:

“What advantages and disadvantages arise from which aspects and what amount of involvement of which types of caregivers in which types of interventions at which treatment phase for children and adolescents of which age and which types of traumatic experiences?”

This provides an excellent starting point for further research: Future empirical studies should assess and investigate additional potential moderators of these interventions’ effectiveness to enable individualized treatment recommendations.

## Supplementary Information

Below is the link to the electronic supplementary material.

Supplementary file1 (DOCX 42 kb)

Supplementary file2 (DOCX 20 kb)

Supplementary file3 (DOCX 381 kb)

Supplementary file4 (DOCX 88 kb)

## Data Availability

The data that support the findings of this study are available from the corresponding author upon request.
