# Supplementary material for: Interventions Involving Caregivers for Children and Adolescents Following Traumatic Events: A Systematic Review and Meta-Analysis
Source: Clin Child Fam Psychol Rev. 2022 Sep 26;26(1):17–32. doi: 10.1007/s10567-022-00415-2 (PMC9879828; doi:10.1007/s10567-022-00415-2)
Supplement: Supplementary file 1 — Supplementary file1 (DOCX 42 kb) [file 10567_2022_415_MOESM1_ESM.docx]

**Supplemental material 1**

**Tables S1-S5**

Table S1

*Search term*

| Population |  | Intervention |  | Control |  | Outcome |  | Study Design |
| --- | --- | --- | --- | --- | --- | --- | --- | --- |
| Child*  Pre-school*  Preschool*  School-child*  Schoolchild*  Schoolage*  Pre-teen  Teen*  Adolescen*  Young adult*  Youth*  Young person*  Young people | AND | Treatment  Therap*  Psychotherap*  Intervention* | AND | Parent*  Mother*  Father*  Parental  Grandparent*  Grandfather*  Grandmother*  Caregiver*  Care-giver*  Carer*  Guardian*  Care stuff  Family  Familial  Families | AND | Trauma*  Traumatic event  Traumatized  Traumatised  Adverse childhood experience*  Child abuse  Child maltreatment  Sexual abuse  Abuse  Maltreatment  Posttraumatic stress disorder*  PTSD  Post traumatic stress disorder*  Post-traumatic stress disorder*  Trauma related disorder*  Trauma-related disorder*  Adjustment disorder*  Complex trauma disorder*  Complex ptsd  Complex posttraumatic stress disorder*  Complex post traumatic stress disorder*  Complex post-traumatic stress disorder*  Mental disorder* following trauma*  Disorder following trauma*  Following trauma*  Event-related | AND | Randomized controlled trial*  Randomised controlled trial*  Efficacy trial*  Quasi randomized controlled trial*  Quasi randomised controlled trial*  Quasi-randomized controlled trial*  Quasi-randomised controlled trial*  Systematic review*  NOT^1^  Case control  Pilot stud*  Cohort stud* |

*Note.* Within a column, OR terms were used. ^1^: Limitations of study design were only applied within the ERIC database. Within the PubMed database, results were restricted to English and German language publications.

Table S2

*Study characteristics*

| Study | Age in years (Range, *M* ± *SD*) | Female (%) | Ethnicity/Migration background (%) | Caregiver involved (%) | Type of trauma (%) | Diagnoses (%) | Intervention (*n*)  Control (*n*) | Caregiver Baseline Psycho-pathology | Caregiver Involvement |
| --- | --- | --- | --- | --- | --- | --- | --- | --- | --- |
| Berkowitz et al., 2011 | 7 – 17, N/A | 52 | Caucasian (32.0), African-American (37.0), Hispanic (22.0), Multiethnic (7.0), Other ethnicities (2.0) | Mothers (90.0), Fathers/Stepfathers (10.0) | N/A | N/A | CFTSI (53)  TAU (53) | N/A | N/A |
| Cohen, & Mannarino, 1996, FU: Cohen, & Mannarino, 1997 | 2 – 7, 4.7 ± N/A | 58 | Caucasian (54.0), African-American (42.0), Other (4.0) | Biological parents (75.0), Adoptive parents (3.0), Grandparents (4.0), Other relatives (3.0), Foster parent (13.0), Other (2.0) | Sexual abuse (100.0) | N/A | CBT-SAP (39)  NST (28) | N/A | N/A |
| Cohen, & Mannarino, 1998 | 7 – 15, 11.1 ± N/A | 69 | Caucasian (59.0), African-American (37.0), Hispanic (2.0), Biracial (2.0) | Both biological parents (10.0), Mother/stepfather (6.0), Father/stepmother (4.0), Adoptive parents (4.0), Mother/boyfriend (4.0), Mother only (33.0), Father only (6.0), Stepfather only (2.0), Grandparents (2.0), Other relatives (4.0), Foster parents (2.0), Other/missing (21.0) | Sexual abuse (100.0) | N/A | SAS-CBT (30)  NST (19) | N/A | N/A |
| Cohen, Deblinger, Mannarino, & Steer, 2004, FU: Deblinger et al., 2006 | 8 – 14, 10.8 ± N/A | 79 | White (60.0), African-American (28.0), Hispanic American (4.0), Biracial (7.0), Other (1.0) | Biological mother (78.0), Adoptive mother (3.0), Stepmother (2.0), Foster mother (4.0), Grandmother (5.0), Other female relative (4.0), Biological father (4.0), Stepfather (1.0), Grandfather (1.0) | Sexual abuse (100.0) | PTSD (89.0) | Tf-CBT (114)  CCT (115) | BDI-II:  Tf-CBT: *M* = 17.3, *SD* = 11.3, *N* = 83  CCT: *M* = 16.1, *SD* = 11.1, *N* = 83;  PERQ:  Tf-CBT: *M* = 51.4, *SD* = 12.5, *N* = 88  CCT: *M* = 49.5, *SD* = 11.4, *N* = 91 | N/A |
| Cohen, Mannarino, & Iyengar, 2011 | 7 – 14, 9.6 ± 2.5 | 50.8 | White (55.6), Black (33.1), Biracial (11.3) | Mothers (100.0) | Domestic violence (100.0) | PTSD (66.7) | Tf-CBT (64)  CCT (60) | N/A | N/A |
| Cohen, Mannarino, & Knudsen, 2005 | 8 – 15, N/A | 68.3 | Caucasian (60.0), African-American (37.0), Biracial (2.0), Hispanic (1.0) | Non-offending parent or primary caregiver^1^ (100.0) | Sexual abuse (100.0) | N/A | Tf-CBT (41)  NST (41) | N/A | N/A |
| Danielson et al., 2012 | 13 – 17, 14.8 ± 1.5 | 88 | African-American (46.0), Caucasian (37.5), Native American (4.2), Biracial (8.3), Hispanic (4.0) | Biological parents (72.6), Other family members (17.1), Non-familial guardians (10.3) | Sexual assault (100.0) | N/A | RR-FT (15)  TAU (15) | N/A | N/A |
| Danielson et al., 2020^0^ | 13 – 18, 15.4±1.3 | 87.1 | White (62.9), Black (29.0), Latino (5.6), Biracial (2.4), Hispanic (4.8) | Mother (58.9), Father (10.5), Grandmother (8.1), Aunt (1.6), Foster mother (7.3), Stepmother (0.8), Case worker (12.1) | Sexual abuse (68.5), Learned about violent death/serious injury of loved one (51.6), Community violence (39.5), Domestic violence (36.3), Physical abuse (33.9), Physical assault (28.2), Natural disaster (21.0), Serious accident (14.5), Medical trauma (13.7), Saw a dead body (11.3), war/political violence (2.4), Other (31.5) | GAD (11.4), Panic disorder (20.2), MDE (16.2), Dysthymic disorder 14.3), Manic episode (1.0), Hypomanic episode (4.8), ODD (25.7), CD 26.5) | RR-FT (61)  TAU (63) | N/A | N/A |
| Dawson et al., 2018 | 7 – 14, 10.4 ± N/A | 48.4 | N/A | N/A | War: separation from caregivers (37.4), witness someone being killed or injured (29.3), family member killed (35.4), close friend killed (32.3) | N/A | Tf-CBT (32)  PS (32) | N/A | N/A |
| Deblinger et al., 1996, FU: Deblinger et al., 1999 | 7 – 13, 9.9 ± 2.0 | 83 | Caucasian (70.0), African-American (21.0), Hispanic (7.0), Other (2.0) | Non-offending mothers (100.0) | Sexual abuse (100.0) | PTSD (71.0), MDD (29.0), ODD (30.0), ADHD (20.0), Separation anxiety (11.0), Overanxious (10.0), CD (6.0), Specific Phobia (5.0), OCD (1.0) | CBT-CP (25)  CBT-C (25) | N/A | N/A |
| Deblinger et al., 2011, FU: Mannarino et al., 2012 | 4 – 11, 7.7 ± 2.1 | 61 | Caucasian (65.0), African-American (14.0), Hispanic (7.0), Other (14.0) | Primary caregiver, Biological mother (84.0) | Sexual abuse (100.0) | N/A | Tf-CBT16+TN (52)  Tf-CBT16-TN (54) | N/A | N/A |
| Diehle et al., 2015 | 8 – 18, 12.9 ± 3.5 | 62 | Dutch (77) | N/A | Accidents (23), sexual assault (17); threat with weapon (13), kidnapping (10), serious illness (7), other single event (30), domestic violence (44), sexual assault (39), other (17) | PTSD (33.3) | Tf-CBT (23)  EMDR (25) | N/A | N/A |
| Dorsey et al., 2020 | 7 – 13, 10.6 ± 1.6 | 50 | N/A | N/A | Death of one or both parents (100.0) | N/A | Tf-CBT (320)  UC (320) | N/A | N/A |
| Goldbeck et al., 2016, FU: Tutus et al., 2017 | 7 – 17, 13.0 ± 2.8 | 71.7 | Whole sample: German native (89.9)  Intervention group: German native (89.5) | Whole sample:  Mother (81.4) Intervention group: Mother (84.2)  Waitlist condition:  Mother (78.6) | Interpersonal (76.7), Accident (23.3) | PTSD (75.5), MDD (19.5), Anxiety Disorder (10.1), ADHD (6.3), CDD/OD (4.4) | Tf-CBT (76)  WL (83) | PDS:  Mild or no symptoms (41.6), Moderate symptoms (24.8), Moderate to severe symptoms, (20.4) Severe symptoms (3.5)  BDI-II:  No or minimal symptoms (67.3),  Mild symptoms (16.8), Moderate symptoms (9.7), Severe symptoms (6.2) | 71.9% of children shared their trauma narrative in a conjoint session with parent(s) |
| Hitchcock et al., 2022 | 3 – 8, 6.3±1.8 | 51.35 | N/A | For CBT-3M: Mother (94.4) | Single incident trauma: Burn (2.7), injury (24.3), acute medical emergency (5.4), home invasion (10.8), road traffic accident (8.1), interpersonal threat to life/injury (24.3), witness event with threat to life/injury of a family member (24.3) | PTSD (100.0) according to DSM-5-  PTSD-YC criteria | CBT-3M (18)  TAU (19) | N/A | N/A |
| Jaberghaderi et al., 2019 | 8 – 12, 10.6 ± 0.9 | 49.6 | Kurdish (100.0) | Both parents (87.3), Grandparents (3.9), Single mothers (4.9), remarried mothers (2.0) | Domestic violence (100.0) | N/A | AF-CBT (40)  EMDR (40) | N/A | N/A |
| Jaycox et al., 2010 | N/A, 11.6 ± 1.4 | 55.9 | Caucasian (48.0), African-American (46.0), Hispanic (5.0), Other (2.0) | Parents^1^ (100.0) | Hurricane (100.0) | PTSD (23.0 in Tf-CBT Condition) | Tf-CBT (60)  CBT-Schools (58) | N/A | N/A |
| Jensen et al., 2014, FU: Aas et al., 2019 | 10 – 18, 15.1 ± 2.2 | 79.5 | Norwegian (73.7), Asian (10.9), One parent Norwegian (8.3), Western European countries (1.3), Eastern European countries (1.3), African countries (1.9), South/Central American countries (1.3), Nordic countries (0.6), Other (0.6) | Mother (71.9), Father (17.2), Foster parents (8.6), Other (2.3) | Sudden death/severe illness of a close person (60.9), violence or threats outside the family (59.0), physical abuse within the family (45.5), witnessing violence within the family (42.9), witnessing violence outside the family (27.6), sexual abuse outside the family (27.6), severe accident (20.5), medical procedure (16.0), robbery/assault (10.9), sexual abuse within the family (7.7), natural disaster (5.8), kidnapping (5.1), other (30.8) | PTSD (66.7) | Tf-CBT (79)  TAU (77) | N/A | N/A |
| Jouriles et al., 2001^0^ | 4 – 9, 5.7 ± 1.9 | 27.7 | African-American (30.6),  Caucasian (27.8), Latino (33.3),  Asian (2.8),  Other (5.5) | Mothers (100.0) | Domestic violence (100.0) | ODD (72.2)  CD (27.7) | MFT (18)  TAU (18) | SCL-90-R:  MFT: *M* = 67.8, *SD* = 8.9  TAU: *M* = 67.4, *SD* = 9.5 | N/A |
| Kassam-Adams et al., 2011 | 8 – 17, 11.5 ± 2.5 | 40 | White (57.6), Black (35.3), Other (7.1) | Parents^1^ (100.0) | Accidents (100.0) | PTSD (21.2) | SPC (46)  TAU (39) | N/A | N/A |
| Kataoka et al., 2003 | N/A, 11.4 ± 1.7 | 50 | N/A | N/A | Community violence (100.0) | N/A | MHP for Immigrants (182)  WL (47) | N/A | Parents attending at least one session: 37% |
| King et al., 2000 | 5 – 17, 11.4 ± N/A | 69 | N/A | N/A | Sexual abuse (100.0) | PTSD (69.0), Dysthymia (19.4), ODD (19.4), Separation Anxiety Disorder (16.7), GAD (13.9), CD (8.3), MDD (5.6), ADHD (5.6), Specific phobia (5.6) | Family CBT (12)  Child CBT (12) | N/A | N/A |
| Kolko, 1996 | 6 – 13, 8.6 ± 2.2 | 27.7 | African-American (47.0), Caucasian (47.0), Biracial (6.0) | Parents^1^ (63% lived with biological mothers), biological parents were the perpetrators for 87% | Physical abuse (100.0) | N/A | FT (18)  RCS (12) | BSI:  FT: *M* = 0.7, *SD* = 0.6  RCS: *M* = 0.8, *SD* = 0.8;  BDI:  FT: *M* = 14.4, *SD* = 9.3  RCS: *M* = 16.6, *SD* = 11.2;  GAS:  FT: *M* = 69, *SD* = 9.8  RCS: *M* = 71,3, *SD* = 11.2 | Attendance at sessions:  Clinic: *M* = 11.7  Home: *M* = 5.0 |
| Konanur et al., 2015^0^ | 6 – 13, N/A | 70.8 | European-Canadian (39.3), African/Caribbean-Canadian (17.9), Asian Canadian (11.6), Latin American-Canadian (10.7), South Asian Canadian (6.3), Aboriginal (1.8), Other (12.5) | Biological mothers (82.6) | N/A | N/A | Tf-CBT (83)  WL (30) | N/A | N/A |
| Langley et al., 2015 | 5 – 11, 7.7 ± 1.4 | 50 | African-American (17.6), Asian (1.4), Caucasian (27.0), Hispanic (48.7), African American/Hispanic (1.3), Asian/Caucasian (1.4), Hispanic/Caucasian (2.7) | Parents^1^ (100.0) | Witness of family member arrested (30.6), witness of violence (26.4), physical violence (24.7), witness of neighborhood violence (24.7), separation from parents (21.9), witness of serious accident (17.8), threat of violence (17.8), close person sick or badly hurt (16.4), close person ill or hospitalized (15.1), close persons death (13.9), serious accident (13.7), animal attack (11.1), threat of robbery (11.0), drug abuse by parent (9.6), other (9.6), witness weapon violence (6.9), witness threat of violence (6.9), kidnap of child or close person (4.2), homelessness or extreme poverty (4.1), attacked with weapon (2.7), natural disaster (1.4) | N/A | Bounce Back (36)  WL (38) | N/A | Parents attending at least one session: 97% |
| Love et al., 2019 | 1 – 5, 3.3 ± 1.1 | 31.3 | African-American (42.2)  Multiracial (28.1)  Latina/Hispanic (18.8)  European-American (10.9) | Mothers (57.8)  Both parents (18.8)  Foster parents (15.6)  Other relatives (7.8) | Separation from primary caregiver (75), witnessing violence in the home (53.1), incarceration of a family member (35.9), witnessing verbal abuse in the home (32.8), experiencing physical abuse (28.1), experiencing neglect (26.6), witnessing community violence (23.4), having a life-threatening illness or injury (21), experiencing verbal abuse (17.2), Other (34.4) | PTSD (100) | EPP (32)  WL (32) | N/A | Parents were required to attend at least five sessions |
| Meiser-Stedman et al., 2017 | 8 – 17, 13.3 ± 2.5 | 72.4 | White British (86.2), Minority (13.8) | Mothers (78.6), fathers (14.3), grandmothers (7.1) | Motor vehicle collisions (51.7), assaults (24.1), medical emergencies (3.4), house fires (3.4), others (17.2) | PTSD (100) | CT-PTSD (14)  WL (15) | N/A | N/A |
| Murray et al., 2015 | 5 – 18, 13.7 ± N/A | 49.9 | Ngoni (21.4), Bemba (31.5), Other (46.3) | Mother (24.5), Father (10.9), None/Other (64.6) | Hit, punched, or kicked at home; Witnessed domestic violence; Heard about violent death of loved one; Beaten up, shot at, or threatened; Witnessed violence; Saw dead body; Saw drowning; Received; painful medical treatment, Experienced disaster, Sexually abused, Injured (N/A) | N/A | Tf-CBT (131)  TAU (126) | N/A | Parents attending at least one session: 57% |
| Nixon et al., 2012, FU: Nixon et al., 2017 | 7 – 17, 10.8 ± 3.0 | 36.4 | Caucasian (84.8), European (6.1), Middle Eastern (3.0), Aboriginal (3.0), Other (3.0) | Parents^1^ (100.0) | Car accident (33.3), injuries (21.2), house fire (12.1), burglary (12.1), others (21.2) | PTSD (64.7) | Tf-CBT (17)  CT (17) | PDS:  Tf-CBT: *M* = 18.8, *SD* = 16.35, *N* = 17  CT: *M* = 14.7, *SD* = 15.0, *N* = 16;  BDI-II:  Tf-CBT: *M* = 12.9, *SD* = 13.8, *N* = 17  CT: *M* = 12.8, *SD* = 12.3, *N* = 16 | N/A |
| O’Callaghan et al., 2013 | 12 – 17, 16.02 ± N/A | 100.0 | Congolese (100.0) | N/A | Inappropriate sexual touch (92), lack of food or water (83), severe punishment (77), threats (75), see a lot of blood/corpses (73), rape (71), carrying heavy loads (65), looting (63), serious illness (62), being badly beaten (60), fights/attacks (56), road accident (54), explosion or fire (46), unplanned pregnancy (46), parental separation/divorce (40), murder (40), forced to lie on a cement floor and soaked with water (40), parental death (38), mutilation (37), burned (27), ambush (23), shot with a bullet (21), abduction (19) | N/A | Tf-CBT (24)  WL (28) | N/A | Attendance at sessions: 82-100% |
| Overbeek et al., 2013 | 6 – 12, 9.2 ± 1.5 | 44.5 | Dutch (43.2), Turkish/Moroccan (18.7), Antilles/Suriname (20.0), Other (18.1) | Mother (95.5) | Domestic violence (100.0) | N/A | IMTN-PP (108)  NSFIP (56) | N/A | N/A |
| Roos et al., 2011 | 4 – 18, 10.1 ± N/A | 44.2 | N/A | Parents^1^ (100.0) | Explosion (100.0) | PTSD (17.3) | CBT (26)  EMDR (26) | N/A | Attendance at sessions:  CBT: *M* = 3.6, *SD* = 1.3  EMDR: *M* = 3.1, *SD* = 1.5 |
| Salloum et al., 2016 | 3 – 7, 5.0 ± 1.5 | 49.1 | American Indian/Alaskan Native (1.9), African-American (26.4), White (64.2), Mixed (7.5) | Biological mother (86.8), Biological father (5.7), Grandmother (3.8), Great aunt (1.9), Aunt (1.9) | Sexual abuse (34), Domestic violence (34), Death/grief (11.3), Physical abuse (3.8), Accidents (5.7), Community violence (1.9), Crime (1.9), Witnessed parent arrest (1.9), Removal from parent/home (3.8), Illness/medical (1.9) | PTSD (43.4) | SC-Tf-CBT (35)  Tf-CBT (18) | SCID-RV PTSD:  SC-Tf-CBT: 42.9%  Tf-CBT: 72.2%;  SCID-RV MDD:  SC-Tf-CBT: 17.2%  Tf-CBT: 22.2% | N/A |
| Santiago et al., 2018 | N/A, 7.8 ± 0.9 | 36.5 | African-American/Black (3.8), White/Caucasian (5.9), Latino (55.8), Latino/Caucasian (23.1), Latino/Native American (5.9), N/A (5.9) | Parents^1^ (83.0 female) | Separation from close person (68.8), family member arrest or deportation (63.0), witness of physical fight (60.4), family member with serious illness (54.3), serious accident (50.0) | N/A | Bounce Back (25)  WL (27) | N/A | Parents attending at least one session: 84% |
| Scheeringa et al., 2011 | 3 – 6, 5.3 ± 1.1 | 33.8 | Black (59.5), White (35.1), Other (5.4) | Primary maternal caregivers^1^ (100.0) | Acute injury (24.0), Witnessed domestic violence (24.0), Hurricane (52.0) | PTSD (24.0) | Tf-CBT (51)  WL (24) | N/A | N/A |
| Schottelkorb et al., 2012 | 6 – 13, 9.2 ± 2.0 | 45.2 | Africa (67.7), Middle East (16.1), Asia (9.7), Europe (6.5) | Parents^1^ (100.0) | N/A | PTSD (48.4) | Tf-CBT (17)  CCPT (14) | N/A | N/A |
| Swart et al., 2014^0^ | 15 – 17, N/A | N/A | African American (48.4), European American (45.1), Hispanic (6.6) | N/A | N/A | CD (58.2), ODD (27.0), PTSD (45.1), MDD (7.4), Anxiety disorder (4.9) | RMDT (61)  TAU (61) | N/A | N/A |

*Note.* ^0^: Included in qualitative analysis only. ^1^: No information if/how many biological or foster parents. ADHD: Attention Deficit Hyperactivity Disorder. AF-CBT: Abuse-Focused Cognitive-Behavioral Therapy. BDI: Beck Depression Inventory. BSI: Brief Symptom Inventory. CBT: Cognitive-Behavioral Therapy. CBT-C: Experimental Cognitive Behavioral Intervention Child Only. CBT-CP: Experimental Cognitive Behavioral Intervention Child And Parent. CBT-SAP: Cognitive-Behavioral Therapy For Sexually Abused Preschool Children. CBT-Schools: Cognitive-Behavioral Intervention for Trauma in Schools. CCPT: Child-Centered Play Therapy. CCT: Child Centered Therapy. CD: Conduct Disorder. CFTSI: Child And Family Traumatic Stress Intervention. CT: Cognitive Therapy. CT-PTSD: Cognitive Therapy for PTSD. EPP: Early Pathways Program. FMDT: Family Mode Deactivation Therapy. FT: Family Therapy. FU: Follow-up. GAD: Generalized Anxiety Disorder. GAS: Global Assessment of Functioning Scale. GT: Group Therapy. IMTN-PP: It's My Turn Now! Psychoeducation Program. IPT: Individual Psychodynamic Therapy. MDD: Major Depressive Disorder. MFT: Multicomponent Family Therapy. MHP For Immigrants: Mental Health For Immigrants Program. NSFIP: Non-Specific Factors Intervention Program. NST: Non-Directive Supportive Therapy. OCD: Obsessive Compulsive Disorder. ODD: Oppositional Defiant Disorder. PDS: Posttraumatic Stress Diagnostic Scale. PERQ: Parent Emotional Reaction Questionnaire. PS: Problem Solving. PTSD: Posttraumatic Stress Disorder. RCS: Routine Community Services. RR-FT: Risk Reduction Through Family Therapy. SAS-CBT: Sexual-Abuse Specific Cognitive Behavioral Therapy. SCID-RV: Structured Clinical Interview for DSM-IV-TR. SCL-90-R: Symptom Checklist-90-Revised. SC-Tf-CBT: Stepped-Care Trauma-Focused Cognitive-Behavioral Therapy. SPC: Stepped Preventive Care. TAU: Treatment as Usual. Tf-CBT: Trauma-Focused Cognitive-Behavioral Therapy. Tf-CBT16+TN: 16-Sessions Tf-CBT With Trauma-Narrative. Tf-CBT16-TN: 16-Session Tf-CBT Without Trauma-Narrative. UC: Usual Care. WL: Waitlist.

Table S3

*Risk of Bias assessment*

| Study | Sequence Generation | Allocation concealment | Blinding of participants and personnel | Blinding of outcome assessment | Incomplete outcome data | Selective outcome reporting | Other sources of bias | Overall |
| --- | --- | --- | --- | --- | --- | --- | --- | --- |
| Berkowitz et al., 2011 | Low risk: ten-subject block design using number containers | Unclear risk: No information available | High risk: Therapists and parents knew about assignment | High risk: unblinded interviewers | Unclear risk: Mixed effects modeling | Low risk: study registry NCT0103165 | Low risk: No other sources of bias apparent | High |
| Cohen, & Mannarino, 1996 | Low risk: Efron's biased coin toss | Unclear risk: Insufficient reporting | High risk: Therapists and participants know about assignment, parents stated being pleased with assignment | Unclear risk: no blinding of caregiver, unclear for study personnel | High risk: Removal of patients with persistent symptoms, differential dropout | Unclear risk: No study protocol available | Low risk: No other sources of bias apparent | High |
| Cohen, & Mannarino, 1998 | Low risk: Efron’s biased coin toss | Unclear risk: Insufficient reporting | High risk: Therapists and parents knew about assignment | Unclear risk: No blinding of participants, no information about personnel | High risk: Removal of patients with persistent symptoms, high and imbalanced dropout rates | Unclear risk: No study protocol | Low risk: No other sources of bias apparent | High |
| Cohen, Deblinger, Mannarino, Steer, 2004 | Unclear risk: Insufficient reporting | Unclear risk: Insufficient reporting | High risk: Therapists and participants knew about assignment | Low risk: Independent blind evaluators | Low risk: ITT analysis | Unclear risk: No study protocol | Low risk: No other sources of bias apparent | High |
| Cohen, Mannarino, & Iyengar, 2011 | Low risk: computer-generated random number series | Low risk: Randomization lists were locked in therapists’ offices | High risk: No blinding | Low risk: Blinded assessor | Low risk: ITT analysis | Unclear risk: Study protocol (NCT00183326) without report of specific outcome instruments | High risk: High dropout | Medium |
| Cohen, Mannarino, & Knudsen, 2005 | Low risk: Random number series generated by computer | Unclear risk: Insufficient reporting | High risk: Therapists and participants knew about assignment | Low risk: Independent blind evaluators | High risk: ITT analysis, but differential dropout rates and removal in case of ongoing behavior problems | Unclear risk: No study protocol available | Low risk: No other sources of bias apparent | High |
| Danielson et al., 2012 | Low risk: computerized blocked randomization | Unclear risk: Insufficient reporting | Unclear risk: Insufficient reporting | Low risk: Blinded assessor | Unclear risk: Insufficient reporting | Unclear risk: No study protocol | Low risk: No other sources of bias apparent | High |
| Danielson et al., 2020 | Low risk: Urn randomization | Unclear risk: Insufficient reporting | High risk: No blinding | Low risk: Blinded assessors | Low risk: ITT analysis | Low risk: NCT01751035, Only secondary outcome not reported | Low risk: No other sources of bias apparent | Low |
| Dawson et al., 2018 | Unclear risk: Insufficient reporting | Unclear risk: Insufficient reporting | High risk: No blinding | Low risk: Blinded assessors | Low risk: ITT | High risk: ACTRN12611000080921, primary outcome not reported | Low risk: No other sources of bias apparent | High |
| Deblinger et al., 1996 | Unclear risk: No information available | Unclear risk: No information available | High risk: Therapists and parents knew about assignment | Unclear risk: Insufficient reporting | Unclear risk: Insufficient information on dropouts | Unclear risk: No study protocol available | Low risk: No other sources of bias apparent | High |
| Deblinger et al., 2011 | Unclear risk: Insufficient reporting | Unclear risk: Insufficient reporting | High risk: Therapists and participants knew about assignment | Low risk: Blinded interviewer | Low risk: ITT analysis | Unclear risk: No study protocol | Low risk: No other sources of bias apparent | High |
| Diehle et al., 2015 | Low risk: block randomization stratified by age | Unclear risk: Insufficient reporting | High risk: Participants and therapists knew about assignment | Low risk: blind assessors | Low risk: ITT analysis | Low risk: study registry (NTR1814) | Low risk: No other sources of bias apparent | Low |
| Dorsey et al., 2020 | Unclear risk: Insufficient reporting | Low risk: Randomization number at coordinator only | High risk: No blinding | Low risk: Blind assessors | Low risk: ITT | Low risk: NCT01822366 | Low risk: No other sources of bias apparent | Low |
| Goldbeck et al., 2016 | Low risk: block randomization with permuted blocks | Low risk: random sequence was hidden | High risk: No blinding | Low risk: Blinded assessors | Low risk: ITT | Low risk: NCT01516827 | Low risk: No other sources of bias apparent | Low |
| Hitchcock et al., 2022 | Low risk: Stratified randomization | Low risk: Informed via email | High risk: No blinding | Low risk: Blinded assessors | Low risk: ITT analysis | Low risk: ISRCTN35018680 | Low risk: No other sources of bias apparent | Low |
| Jaberghaderi et al., 2019 | Low risk: Picking names out of hat | Unclear risk: Insufficient reporting | High risk: No blinding | Low risk: Blinded assessors | Unclear risk: Insufficient reporting | Unclear risk: No study protocol | Low risk: No other sources of bias apparent | High |
| Jaycox et al., 2010 | Low risk: Randomized within strata | Unclear risk: Insufficient reporting | Unclear risk: Insufficient reporting, probably no blinding | Unclear risk: Insufficient reporting, but probably no blinding | Low risk: ITT analysis | Unclear risk: No study protocol; shift from the original aims of analysis because of low uptake in TF-CBT | High risk: Different symptom inclusion criteria among groups | High |
| Jensen et al., 2014 | Low risk: Computer-generated randomized block procedure | Unclear risk: Insufficient reporting | Low risk: Blinding according to study protocol | Low risk: Blinded assessor according to study protocol | Low risk: ITT analysis | Unclear risk: Study protocol, but no specific reporting of outcome measures | Low risk: No other sources of bias apparent | Low |
| Jouriles et al., 2001 | Unclear risk: Insufficient reporting | Unclear risk: Insufficient reporting | High risk: Therapists and participants knew about assignment | Low risk: Coders were blinded | Unclear risk: Insufficient reporting, no ITT analysis | Unclear risk: No study protocol available | Low risk: No other sources of bias apparent | High |
| Kassam-Adams et al., 2011 | Low risk: Random number list generated by computer | Low risk: Sealed envelope | High risk: No blinding | Low risk: Assessors were blinded | Low risk: ITT analysis | Low risk: study protocol (NCT00451282) | High risk: Differences in recent/ongoing mental health treatment among groups | Low |
| Kataoka et al., 2003 | High risk: Not all were randomized | Unclear risk: Insufficient reporting | High risk: Therapists and participants knew about assignment | High risk: No blinding | High risk: Differential attrition, insufficient reporting of reasons, exclusion of dropouts | Unclear risk: No study protocol available | Low risk: No other sources of bias apparent | High |
| King et al., 2000 | Unclear risk: Insufficient reporting | Unclear risk:  Insufficient reporting | High risk: Therapists and participants knew about assignment | Unclear risk: Insufficient reporting | Low risk: ITT analysis | Unclear risk: No study protocol | Low risk: No other sources of bias apparent | High |
| Kolko, 1996 | High risk: only 2 of 3 conditions were randomized | High risk: for control condition, caseworkers referred | High risk: Differences in expectancy assessment | Unclear risk: Insufficient reporting | Unclear risk: Insufficient information on dropouts | Unclear risk: No study protocol available | Low risk: No other sources of bias apparent | High |
| Konanur et al., 2015 | Unclear risk:  Insufficient reporting | Unclear risk:  Insufficient reporting | High risk: No blinding | High risk: No blinding | Unclear risk:  Insufficient reporting | Unclear risk: No study protocol | Low risk: No other sources of bias apparent | High |
| Langley et al., 2015 | Low risk: standard table of random assignment | Unclear risk: Insufficient reporting | High risk: No blinding | High risk: No blinding | Unclear risk: Mixed-effect models | Unclear risk: No study protocol | Low risk: No other sources of bias apparent | High |
| Love et al., 2019 | Low risk: computer-derived random numbers table | Unclear risk: Insufficient reporting | High risk:  No blinding | High risk: No blinding (therapists = assessors) | Low risk: ITT | Unclear risk: No study protocol | Low risk: No other sources of bias apparent | High |
| Meiser-Stedman et al., 2017 | Low risk: minimization procedure with stratification | Unclear risk: Insufficient reporting | High risk: No blinding | Low risk: Blind assessors | Low risk: ITT analysis | High risk: ISRCTN38352118, switch of primary and secondary outcome | Low risk: No other sources of bias apparent | Medium |
| Murray et al., 2015 | Low risk: Random number generator | Low risk: Sealed envelope | High risk: No blinding | Low risk: Assessors were masked | Low risk: ITT analysis | Low risk: IRB00004157 | Low risk: No other sources of bias apparent | Low |
| Nixon et al., 2012 | Low risk: block randomization | Unclear risk: Insufficient reporting | Unclear risk: Insufficient reporting | Low risk: Blinded assessors | Low risk: ITT | Unclear risk: No study protocol | Low risk: No other sources of bias apparent | Medium |
| O’Callaghan et al., 2013 | Low risk: computer-generated random sequence | Low risk: allocation was concealed from those responsible for participant enrollment | High risk: No blinding of participants possible | Low risk: Blinded assessor | Low risk: ITT analysis | Low risk: NCT01483261 | Low risk: No other sources of bias apparent | Low |
| Overbeek et al., 2013 | Low risk: block randomization | Unclear risk: Insufficient reporting | Unclear risk: Insufficient reporting | Unclear risk: Insufficient reporting | High risk: ITT, but exclusion of cases without data after baseline | Unclear risk: No study protocol | High risk: differential symptoms among groups | High |
| Roos et al., 2011 | Low risk: Coin flipping | Unclear risk: Insufficient reporting | Unclear risk: Insufficient reporting, probably no blinding | Low risk: Independent blinded assessor | Low risk: ITT, Multivariate Imputation by Chained Equations | Unclear risk: No study protocol available | Low risk: No other sources of bias apparent | Medium |
| Salloum et al., 2016 | Low risk: computerized randomized block procedure | Unclear risk: Insufficient reporting | Low risk: No blinding, but no differences in expectations /satisfaction | Low risk: Blinded evaluator | Low risk: ITT | Low risk: NCT01603563 | Low risk: No other sources of bias apparent | Low |
| Santiago et al., 2018 | Low risk: Block randomization of schools | Unclear risk: Insufficient reporting | High risk: No blinding | High risk: No blinding | Low risk: ITT | Unclear risk: No study protocol | Low risk: No other sources of bias apparent | High |
| Scheeringa et al., 2011 | High risk: blocked randomization procedure was interrupted | Unclear risk: Insufficient reporting | High risk: No blinding | Unclear risk: Insufficient reporting | Unclear risk: Random effects regression | Unclear risk: No study protocol | Low risk: No other sources of bias apparent | High |
| Schottelkorb et al., 2012 | Low risk: computer-generated random numbers table | Unclear risk: Insufficient reporting | Unclear risk: Insufficient reporting | High risk: No blinding | High risk: No ITT; differential attrition | Unclear risk: No study protocol | Low risk: No other sources of bias apparent | High |
| Swart et al., 2014 | Unclear risk: Insufficient reporting | Unclear risk: Insufficient reporting | High risk: Patients and therapists knew about assignment | High risk: No blinding | Unclear risk: Insufficient reporting on dropouts, analysis | Unclear risk: No study protocol | Low risk: No other sources of bias apparent | High |

Table S4

*Results of sensitivity analyses*

| Symptoms | Successful Randomization | | | | | | | Intent-to-treat analysis | | | | | | | | | | | | | | | Risk of Bias | | | | | | | | | | | | | | | |
| --- | --- | --- | --- | --- | --- | --- | --- | --- | --- | --- | --- | --- | --- | --- | --- | --- | --- | --- | --- | --- | --- | --- | --- | --- | --- | --- | --- | --- | --- | --- | --- | --- | --- | --- | --- | --- | --- | --- |
|  | *Q* | | *df* | | | *p* | | *Q* | | | | *df* | | | | | *p* | | | | | | *Q* | | | | | | | *df* | | | | | | | | *p* |
| PTSD child report | 0.05 | | 1 | | | .816 | | 0.19 | | | | 1 | | | | | .667 | | | | | | 1.45 | | | | | | | 2 | | | | | | | | .484 |
| PTSD parent report | 2.68 | | 1 | | | .102 | | 0.01 | | | | 1 | | | | .922 | | | | | | 7.27 | | | | | | | 2 | | | | | | | | | **.026** |
| DEP child report | 0.06 | | 1 | | | .799 | | 2.14 | | | | 1 | | | | .144 | | | | | | 0.38 | | | | | | | 2 | | | | | | | | | .828 |
| DEP parent report | 2.29 | | 1 | | | .130 | | 1.68 | | | 1 | | | | .195 | | | | | | | 0.67 | | | | | | | 1 | | | | | | | | | .412 |
| ANX child report | N/A | | | | | | 0.42 | | | 1 | | | | | .515 | | | | | | 2.65 | | | | | | | 2 | | | | | | | | | .266 | |
| ANX parent report | 0.96 | | 1 | | .326 | | 0.96 | | | 1 | | | | .326 | | | | | | | 12.26 | | | | | | | 1 | | | | | | | | | **< .001** | |
| ADHD parent report | 0.26 | | 1 | | .611 | | 0.26 | | | 1 | | | | .611 | | | | | | | 1.48 | | | | | | 2 | | | | | | | | | .477 | | |
| INT child report | N/A | | | | | | 23.45 | | | 1 | | | | **< .001** | | | | | | 3.63 | | | | | | 1 | | | | | | | | | .057 | | | |
| INT parent report | N/A | | | | | | 0.11 | | | 1 | | | | .742 | | | | | | 0.12 | | | | | 2 | | | | | | | | | .943 | | | | |
| EXT child report | N/A | | | | | | 11.54 | | | 1 | | | | **< .001** | | | | | | 2.16 | | | | | 1 | | | | | | | | .142 | | | | | |
| EXT parent report | 1.69 | 1 | | .194 | | | 0.07 | | | 1 | | | | .795 | | | | | 0.37 | | | | | | 2 | | | | | | | .829 | | | | | | |
| BEH child report | N/A | | | | | | 2.62 | | 1 | | | | .106 | | | | | 23.43 | | | | | | 2 | | | | | | | **< .001** | | | | | | | |

Table S5

*Results of subgroup analyses*

| Symptoms | Sex | Age | Symptom severity | Type of traumatic event | Interventions’ trauma focus | Type of caregiver | Control condition | Format of caregiver involvement | Extent of caregiver involvement |
| --- | --- | --- | --- | --- | --- | --- | --- | --- | --- |
| PTSD child report  *F/Q*  *df*  *p* | 5.94  1, 26  **.021** | 2.72  1, 26  .111 | 13.34  2, 10  **.002** | 1.99  3, 21  .147 | 0.26  1, 26  .616 | N/A | 8.04  2  **.018** | 2.43  2  .296 | 1.55  2  .459 |
| PTSD parent report  *F/Q*  *df*  *p* | 0.19  1, 14  .673 | 0.34  1, 14  .571 | 0.57  2, 3  .616 | 0.57  3, 11  .647 | 2.74  1, 14  .119 | N/A | 8.57  2  **.014** | 0.72  2  .697 | 0.01  1  .911 |
| DEP child report  *F/Q*  *df*  *p* | 0.98  1, 19  .335 | 0.46  1, 19  .461 | 6.55  2, 9  **.018** | N/A | 0.38  1, 20  .378 | N/A | 3.55  2  .169 | 1.62  2  .446 | 3.66  2  .160 |
| DEP parent report  *F/Q*  *df*  *p* | 1.09  1, 3  .372 | 1.08  1, 3  .374 | N/A | 1.61  1, 3  .294 | 0.29  1, 3  .627 | N/A | 6.98  1  **.008** | 6.98  1  **.008** | 0.00  1  .952 |
| ANX child report  *F/Q*  *df*  *p* | 0.47  1, 12  .506 | 1.38  1, 12  .263 | 5.91  2, 6  **.038** | 1.36  3, 10  .309 | 0.01  1, 12  .944 | 1.92  2  .382 | 1.68  2  .431 | 1.92  2  .382 | 0.15  1  .696 |
| ANX parent report  *F/Q*  *df*  *p* | 28.69  1, 1  .118 | 10.95  1, 1  .187 | N/A | N/A | 0.27  1, 1  .693 | N/A | N/A | 12.26  1  **< .001** | N/A |
| ADHD parent report  *F/Q*  *df*  *p* | 0.63  1, 1  .573 | 0.26  1, 1  .695 | 68.86  1, 1  .076 | N/A | 68.86  1, 1  .076 | N/A | 0.43  1  .514 | 0.43  1  .514 | 0.43  1  .514 |
| INT child report  *F/Q*  *df*  *p* | 1.38  1, 4  .306 | 1.16  1, 4  .342 | N/A | N/A | 1.28  1, 5  .309 | N/A | 23.45  1  **< .001** | 24.87  2  **< .001** | 3.63  1  .057 |
| INT parent report  *F/Q*  *df*  *p* | 0.03  1, 17  .864 | 0.59  1, 17  .449 | 3.76  2, 7  .078 | 0.13  3, 16  .941 | 0.91  1, 18  .353 | N/A | 4.35  2  .114 | 1.03  2  .597 | 0.01  1  .930 |
| EXT child report  *F/Q*  *df*  *p* | 1.52  1, 4  .285 | 1.55  1, 4  .281 | N/A | N/A | 0.63  1, 5  .464 | N/A | 11.54  1  **< .001** | 12.50  2  **.002** | 2.16  1  .142 |
| EXT parent report  *F/Q*  *df*  *p* | 0.34  1, 16  .568 | 0.01  1, 16  .937 | 2.67  2, 6  .148 | 0.441  3, 15  .727 | 0.678  1, 17  .422 | N/A | 3.83  2  .148 | 0.20  2  .907 | 0.22  1  .642 |
| BEH child report  *F/Q*  *df*  *p* | 4.28  1, 8  .072 | 0.00  1, 8  .989 | N/A | N/A | 0.01  1, 8  .907 | N/A | 0.98  2  .614 | 0.82  2  .662 | 0.81  1  .368 |
