# Supplementary material for: Interventions Involving Caregivers for Children and Adolescents Following Traumatic Events: A Systematic Review and Meta-Analysis
Source: Clin Child Fam Psychol Rev. 2022 Sep 26;26(1):17–32. doi: 10.1007/s10567-022-00415-2 (PMC9879828; doi:10.1007/s10567-022-00415-2)
Supplement: Supplementary file 2 — Supplementary file2 (DOCX 20 kb) [file 10567_2022_415_MOESM2_ESM.docx]

**Supplemental material 2**

**References of included studies**

Aas, E., Iversen, T., Holt, T., Ormhaug, S. M., & Jensen, T. K. (2019). Cost-Effectiveness Analysis of Trauma-Focused Cognitive Behavioral Therapy: A Randomized Control Trial among Norwegian Youth. *Journal of Clinical Child and Adolescent Psychology,* *48*, S298-S311. https://doi.org/10.1080/15374416.2018.1463535

Berkowitz, S. J., Stover, C. S., & Marans, S. R. (2011). The Child and Family Traumatic Stress Intervention: Secondary prevention for youth at risk of developing PTSD. *Journal of Child Psychology and Psychiatry, and Allied Disciplines*, *52*(6), 676–685. https://doi.org/10.1111/j.1469-7610.2010.02321.x

Cohen, J. A., & Mannarino, A. P. (1996). A treatment outcome study for sexually abused preschool children: Initial findings. *Journal of the American Academy of Child & Adolescent Psychiatry, 35*(1), 42–50. https://doi.org/10.1097/00004583-199601000-00011

Cohen, J. A., & Mannarino, A. P. (1997). A treatment study for sexually abused preschool children: Outcome during a one-year follow-up. *Journal of the American Academy of Child and Adolescent Psychiatry*, *36*(9), 1228–1235. https://doi.org/10.1097/00004583-199709000-00015

Cohen, J. A., & Mannarino, A. P. (1998). Interventions for sexually abused children: Initial treatment outcome findings. *Child Maltreatment, 3*(1), 17–26. https://doi.org/10.1177/1077559598003001002

Cohen, J. A., Deblinger, E., Mannarino, A. P., & Steer, R. A. (2004). A multisite, randomized controlled trial for children with sexual abuse-related PTSD symptoms. *Journal of the American Academy of Child & Adolescent Psychiatry*, *43*(4), 393–402. https://doi.org/10.1097/00004583-200404000-00005

Cohen, J. A., Mannarino, A. P., & Iyengar, S. (2011). Community treatment of posttraumatic stress disorder for children exposed to intimate partner violence: A randomized controlled trial. *Archives of Pediatrics & Adolescent Medicine, 165*(1), 16–21. https://doi.org/10.1001/archpediatrics.2010.247

Cohen, J. A., Mannarino, A. P., & Knudsen, K. (2005). Treating sexually abused children: 1 year follow-up of a randomized controlled trial. *Child Abuse & Neglect, 29*(2), 135–145. https://doi.org/10.1016/j.chiabu.2004.12.005

Danielson, C. K., Adams, Z., McCart, M. R., Chapman, J. E., Sheidow, A. J., Walker, J., . . . Arellano, M. A. de (2020). Safety and Efficacy of Exposure-Based Risk Reduction through Family Therapy for Co-occurring Substance Use Problems and Posttraumatic Stress Disorder Symptoms Among Adolescents: A Randomized Clinical Trial. *JAMA Psychiatry*, *77*(6), 574–586. https://doi.org/10.1001/jamapsychiatry.2019.4803

Danielson, C. K., McCart, M. R., Walsh, K., Arellano, M. A. de, White, D., & Resnick, H. S. (2012). Reducing substance use risk and mental health problems among sexually assaulted adolescents: A pilot randomized controlled trial. *Journal of Family Psychology*, *26*(4), 628–635. https://doi.org/10.1037/a0028862

Dawson, K., Joscelyne, A., Meijer, C., Steel, Z., Silove, D., & Bryant, R. A. (2018). A controlled trial of trauma-focused therapy versus problem-solving in Islamic children affected by civil conflict and disaster in Aceh, Indonesia. *The Australian and New Zealand journal of psychiatry, 52*(3), 253–261. https://doi.org/10.1177/0004867417714333

Deblinger, E., Lippmann, J., & Steer, R. (1996). Sexually Abused Children Suffering Posttraumatic Stress Symptoms: Initial Treatment Outcome Findings. *Child Maltreatment*, *1*(4), 310–321. https://doi.org/10.1177/1077559596001004003

Deblinger, E., Mannarino, A. P., Cohen, J. A., Runyon, M. K., & Steer, R. A. (2011). Trauma-focused cognitive behavioral therapy for children: Impact of the trauma narrative and treatment length. *Depression and Anxiety*, *28*(1), 67–75. https://doi.org/10.1002/da.20744

Deblinger, E., Mannarino, A. P., Cohen, J. A., & Steer, R. A. (2006). A follow-up study of a multisite, randomized, controlled trial for children with sexual abuse-related PTSD symptoms. *Journal of the American Academy of Child and Adolescent Psychiatry*, *45*(12), 1474–1484. https://doi.org/10.1097/01.chi.0000240839.56114.bb

Deblinger, E., Steer, R. A., & Lippmann, J. (1999). Two-year follow-up study of cognitive behavioral therapy for sexually abused children suffering post-traumatic stress symptoms. *Child Abuse & Neglect*, *23*(12), 1371–1378. https://doi.org/10.1016/S0145-2134(99)00091-5

Diehle, J., Opmeer, B. C., Boer, F., Mannarino, A. P., & Lindauer, R. J. L. (2015). Trauma-focused cognitive behavioral therapy or eye movement desensitization and reprocessing: What works in children with posttraumatic stress symptoms? A randomized controlled trial. *European Child & Adolescent Psychiatry*, *24*(2), 227–236. https://doi.org/10.1007/s00787-014-0572-5

Dorsey, S., Lucid, L., Martin, P., King, K. M., O'Donnell, K., Murray, L. K., . . . Whetten, K. (2020). Effectiveness of Task-Shifted Trauma-Focused Cognitive Behavioral Therapy for Children Who Experienced Parental Death and Posttraumatic Stress in Kenya and Tanzania: A Randomized Clinical Trial. *JAMA Psychiatry*, *77*(5), 464–473. https://doi.org/10.1001/jamapsychiatry.2019.4475

Goldbeck, L., Muche, R., Sachser, C., Tutus, D., & Rosner, R. (2016). Effectiveness of Trauma-Focused Cognitive Behavioral Therapy for Children and Adolescents: A Randomized Controlled Trial in Eight German Mental Health Clinics. *Psychotherapy and Psychosomatics*, *85*(3), 159–170. https://doi.org/10.1159/000442824

Hitchcock, C., Goodall, B., Wright, I. M., Boyle, A., Johnston, D., Dunning, D., … Dalgleish, T. (2022). The early course and treatment of posttraumatic stress disorder in very young children: diagnostic prevalence and predictors in hospital-attending children and a randomized controlled proof-of-concept trial of trauma-focused cognitive therapy, for 3- to 8-year-olds. *Journal of child psychology and psychiatry, and allied disciplines, 63*(1), 58–67. https://doi.org/10.1111/jcpp.13460

Jaberghaderi, N., Rezaei, M., Kolivand, M., & Shokoohi, A. (2019). Effectiveness of Cognitive behavioral Therapy and Eye Movement Desensitization and Reprocessing in Child Victims of Domestic Violence. *Iranian Journal of Psychiatry*, *14*(1), 67–75.

Jaycox, L. H., Cohen, J. A., Mannarino, A. P., Walker, D. W., Langley, A. K., Gegenheimer, K. L., Scott, M., & Schonlau, M. (2010). Children's mental health care following Hurricane Katrina: a field trial of trauma-focused psychotherapies. *Journal of traumatic stress, 23*(2), 223–231. https://doi.org/10.1002/jts.20518

Jensen, T. K., Holt, T., Ormhaug, S. M., Egeland, K., Granly, L., Hoaas, L. C., Hukkelberg, S. S., Indregard, T., Stormyren, S. D., & Wentzel-Larsen, T. (2014). A randomized effectiveness study comparing trauma-focused cognitive behavioral therapy with therapy as usual for youth. *Journal of Clinical Child and Adolescent Psychology, 43*(3), 356–369. https://doi.org/10.1080/15374416.2013.822307

Jouriles, E. N., McDonald, R., Spiller, L., Norwood, W. D., Swank, P. R., Stephens, N., . . . Buzy, W. M. (2001). Reducing conduct problems among children of battered women. *Journal of Consulting and Clinical Psychology*, *69*(5), 774–785. https://doi.org/10.1037/0022-006X.69.5.774

Kassam-Adams, N., García-España, J. F., Marsac, M. L., Kohser, K. L., Baxt, C., Nance, M., & Winston, F. (2011). A pilot randomized controlled trial assessing secondary prevention of traumatic stress integrated into pediatric trauma care. *Journal of traumatic stress, 24*(3), 252–259. https://doi.org/10.1002/jts.20640

Kataoka, S. H., Stein, B. D., Jaycox, L. H., Wong, M., Escudero, P., Tu, W., Zaragoza, C., & Fink, A. (2003). A school-based mental health program for traumatized Latino immigrant children. *Journal of the American Academy of Child and Adolescent Psychiatry, 42*(3), 311–318. https://doi.org/10.1097/00004583-200303000-00011

King, N. J., Tonge, B. J., Mullen, P., Myerson, N., Heyne, D., Rollings, S., . . . Ollendick, T. H. (2000). Treating sexually abused children with posttraumatic stress symptoms: A randomized clinical trial. *Journal of the American Academy of Child and Adolescent Psychiatry*, *39*(11), 1347–1355. https://doi.org/10.1097/00004583-200011000-00008

Kolko, D. J. (1996). Individual Cognitive Behavioral Treatment and Family Therapy for Physically Abused Children and their Offending Parents: A Comparison of Clinical Outcomes. *Child Maltreatment, 1*(4), 322–342. https://doi.org/10.1177/1077559596001004004

Konanur, S., Muller, R. T., Cinamon, J. S., Thornback, K., & Zorzella, K. P. M. (2015). Effectiveness of Trauma-Focused Cognitive Behavioral Therapy in a community-based program. *Child Abuse & Neglect*, *50*, 159–170. https://doi.org/10.1016/j.chiabu.2015.07.013

Langley, A. K., Gonzalez, A., Sugar, C. A., Solis, D., & Jaycox, L. (2015). Bounce back: Effectiveness of an elementary school-based intervention for multicultural children exposed to traumatic events. *Journal of Consulting and Clinical Psychology*, *83*(5), 853–865. https://doi.org/10.1037/ccp0000051

Love, J. R., & Fox, R. A. (2019). Home-Based Parent Child Therapy for Young Traumatized Children Living In Poverty: A Randomized Controlled Trial. *Journal of Child & Adolescent Trauma*, *12*(1), 73–83. https://doi.org/10.1007/s40653-017-0170-z

Mannarino, A. P., Cohen, J. A., Deblinger, E., Runyon, M. K., & Steer, R. A. (2012). Trauma-focused cognitive-behavioral therapy for children: Sustained impact of treatment 6 and 12 months later. *Child Maltreatment*, *17*(3), 231–241. https://doi.org/10.1177/1077559512451787

Meiser-Stedman, R., Smith, P., McKinnon, A., Dixon, C., Trickey, D., Ehlers, A., … Dalgleish, T. (2017). Cognitive therapy as an early treatment for post-traumatic stress disorder in children and adolescents: a randomized controlled trial addressing preliminary efficacy and mechanisms of action. *Journal of child psychology and psychiatry, and allied disciplines, 58*(5), 623–633. https://doi.org/10.1111/jcpp.12673

Murray, L. K., Skavenski, S., Kane, J. C., Mayeya, J., Dorsey, S., Cohen, J. A., . . . Bolton, P. A. (2015). Effectiveness of Trauma-Focused Cognitive Behavioral Therapy Among Trauma-Affected Children in Lusaka, Zambia: A Randomized Clinical Trial. *JAMA Pediatrics*, *169*(8), 761–769. https://doi.org/10.1001/jamapediatrics.2015.0580

Nixon, R. D., Sterk, J., & Pearce, A. (2012). A randomized trial of cognitive behaviour therapy and cognitive therapy for children with posttraumatic stress disorder following single-incident trauma. *Journal of abnormal child psychology, 40*(3), 327–337. https://doi.org/10.1007/s10802-011-9566-7

Nixon, R., Sterk, J., Pearce, A., & Weber, N. (2017). A randomized trial of cognitive behavior therapy and cognitive therapy for children with posttraumatic stress disorder following single-incident trauma: Predictors and outcome at 1-year follow-up. *Psychological trauma: Theory, research, practice and policy, 9*(4), 471–478. https://doi.org/10.1037/tra0000190

O'Callaghan, P., McMullen, J., Shannon, C., Rafferty, H., & Black, A. (2013). A randomized controlled trial of trauma-focused cognitive behavioral therapy for sexually exploited, war-affected Congolese girls. *Journal of the American Academy of Child and Adolescent Psychiatry*, *52*(4), 359–369. https://doi.org/10.1016/j.jaac.2013.01.013

Overbeek, M. M., de Schipper, J. C., Lamers-Winkelman, F., & Schuengel, C. (2013). Effectiveness of specific factors in community-based intervention for child-witnesses of interparental violence: a randomized trial. *Child abuse & neglect, 37*(12), 1202–1214. https://doi.org/10.1016/j.chiabu.2013.07.007

Roos, C. de, Greenwald, R., den Hollander-Gijsman, M., Noorthoorn, E., van Buuren, S., & Jongh, A. de (2011). A randomised comparison of cognitive behavioural therapy (CBT) and eye movement desensitisation and reprocessing (EMDR) in disaster-exposed children. *European Journal of Psychotraumatology*, *2.* https://doi.org/10.3402/ejpt.v2i0.5694

Salloum, A., Wang, W., Robst, J., Murphy, T. K., Scheeringa, M. S., Cohen, J. A., & Storch, E. A. (2016). Stepped care versus standard trauma‐focused cognitive behavioral therapy for young children. *Journal of Child Psychology and Psychiatry, 57*(5), 614–622. https://doi.org/10.1111/jcpp.12471

Santiago, C. D., Raviv, T., Ros, A. M., Brewer, S. K., Distel, L. M. L., Torres, S. A., . . . Langley, A. K. (2018). Implementing the Bounce Back trauma intervention in urban elementary schools: A real-world replication trial. *School Psychology Quarterly,* *33*(1), 1–9. https://doi.org/10.1037/spq0000229

Scheeringa, M. S., Weems, C. F., Cohen, J. A., Amaya‐Jackson, L., & Guthrie, D. (2011). Trauma‐focused cognitive‐behavioral therapy for posttraumatic stress disorder in three‐through six year‐old children: A randomized clinical trial. *Journal of Child Psychology and Psychiatry, 52*(8), 853–860. https://doi.org/10.1111/j.1469-7610.2010.02354.x

Schottelkorb, A. A., Doumas, D. M., & Garcia, R. (2012). Treatment for childhood refugee trauma: A randomized, controlled trial. *International Journal of Play Therapy, 21*(2), 57–73. https://doi.org/10.1037/a0027430

Swart, J., & Apsche, J. (2014). A comparative treatment efficacy study of conventional therapy and mode deactivation therapy (MDT) for adolescents with conduct disorders, mixed personality disorders, and experiences of childhood trauma. *International Journal of Behavioral Consultation and Therapy*, *9*(1), 23–29. https://doi.org/10.1037/h0101011

Tutus, D., Pfeiffer, E., Rosner, R., Sachser, C., & Goldbeck, L. (2017). Sustainability of treatment effects of trauma-focused cognitive-behavioral therapy for children and adolescents: Findings from 6- and 12-month follow-ups. *Psychotherapy and Psychosomatics, 86*(6), 379–381. https://doi.org/10.1159/000481198
