# Supplementary material for: Interventions Involving Caregivers for Children and Adolescents Following Traumatic Events: A Systematic Review and Meta-Analysis
Source: Clin Child Fam Psychol Rev. 2022 Sep 26;26(1):17–32. doi: 10.1007/s10567-022-00415-2 (PMC9879828; doi:10.1007/s10567-022-00415-2)

**Supplemental material 3**

**Figures S1-S21**

**Figure S1**

*Forest plot for child-reported PTSD symptoms at three to four months*

**Figure S2**


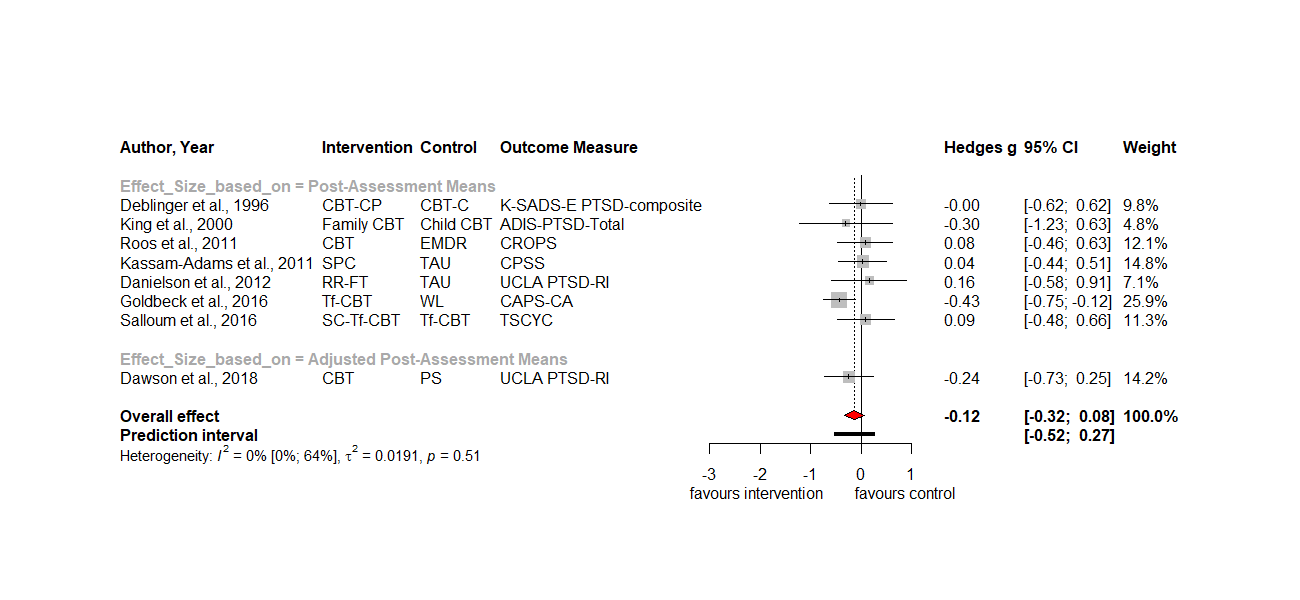


*Forest plot for child-reported PTSD symptoms at six months
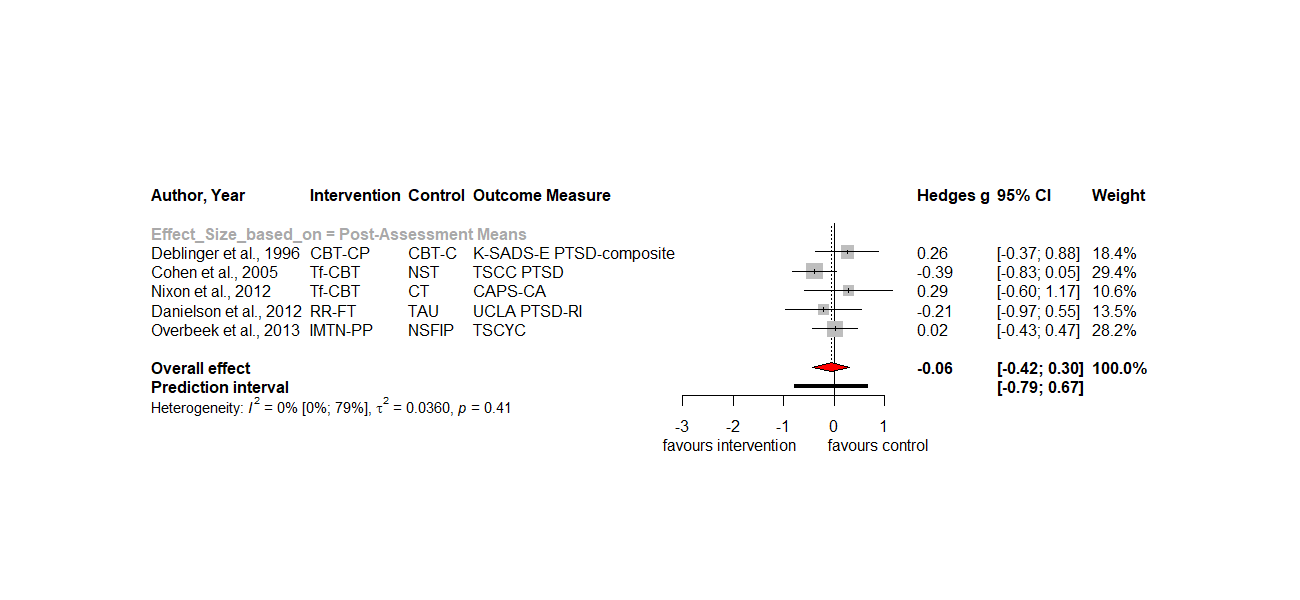
*

**Figure S3**

*Forest plot for child-reported PTSD symptoms at twelve months*


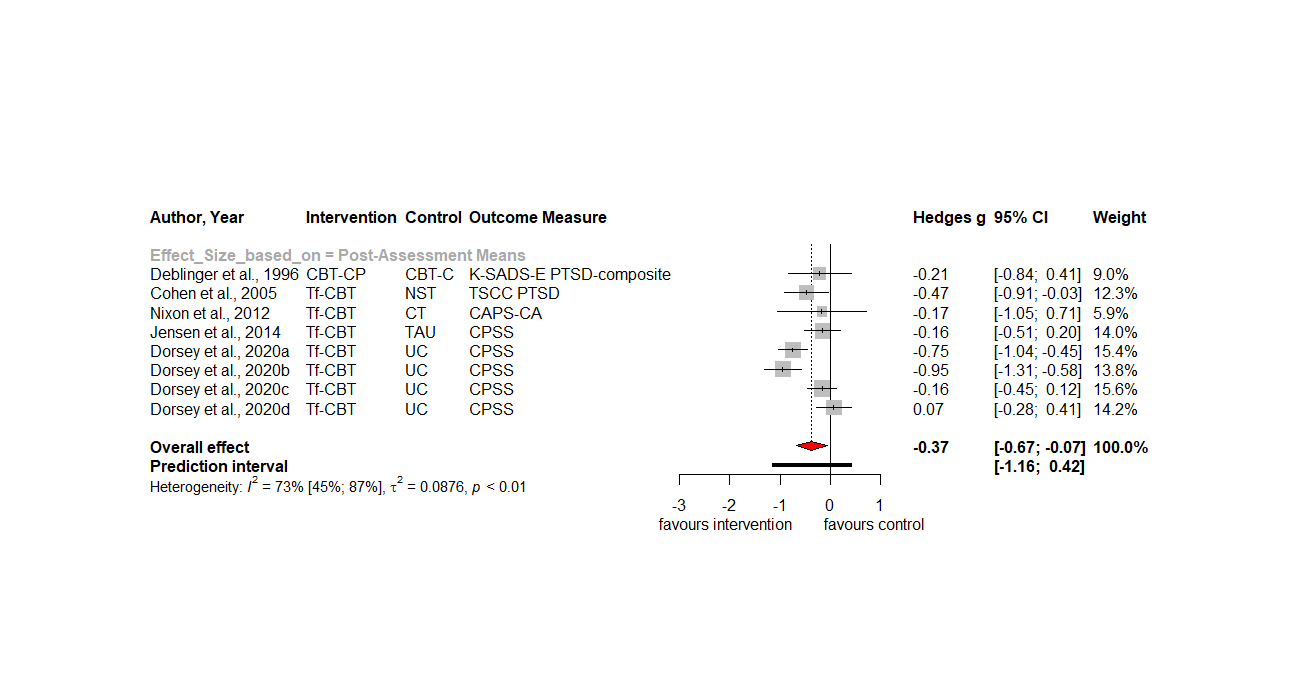


**Figure S4**

*Forest plot for parent-reported PTSD symptoms at three months*


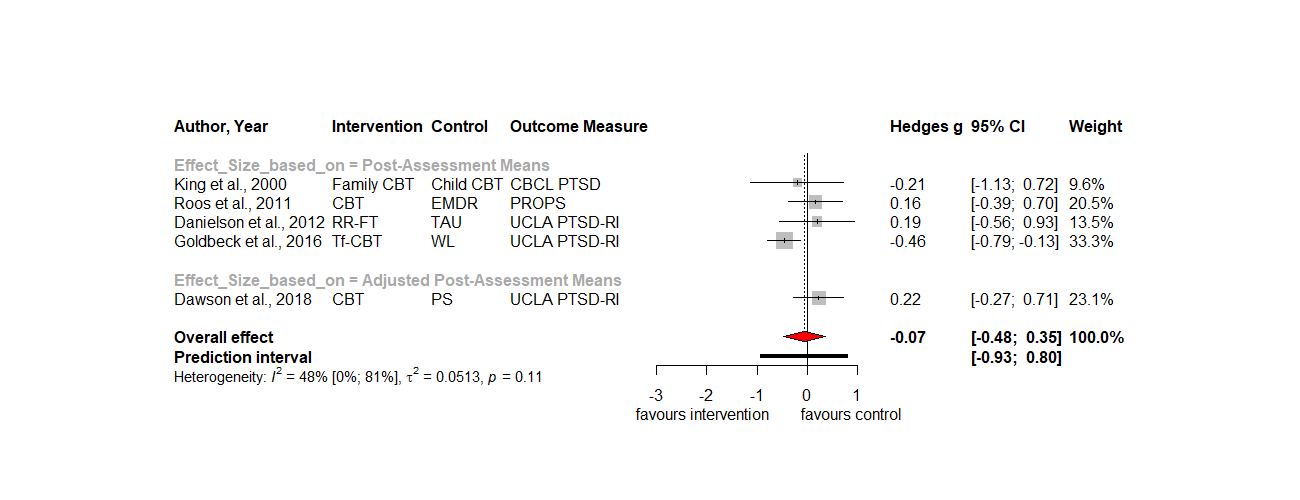


**Figure S5**

*Forest plot for parent-reported PTSD symptoms at twelve months*


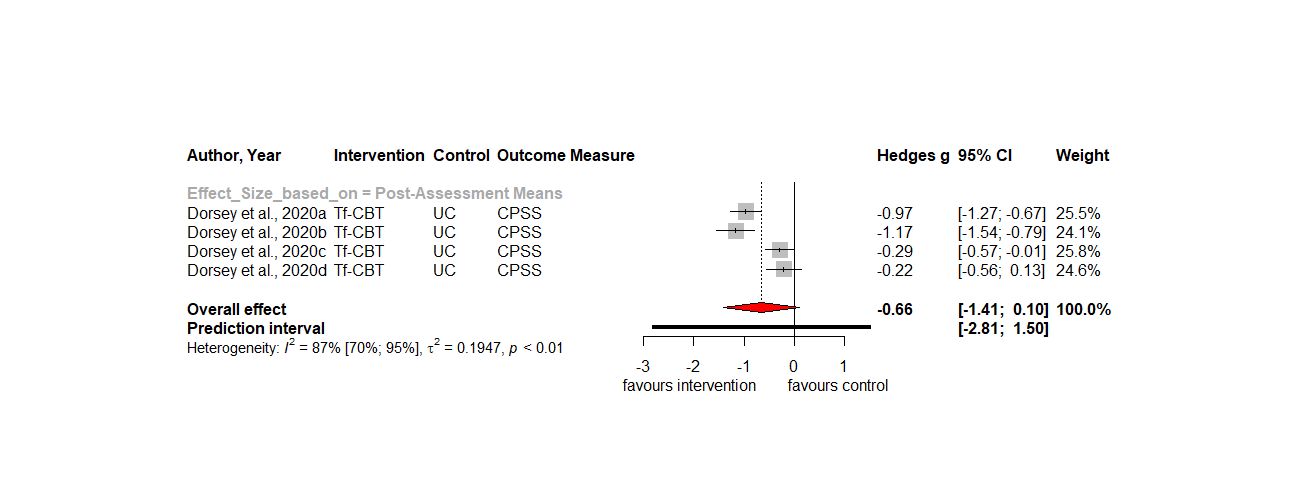


**Figure S6**

*Forest plot for child-reported depressive symptoms at three to four months*


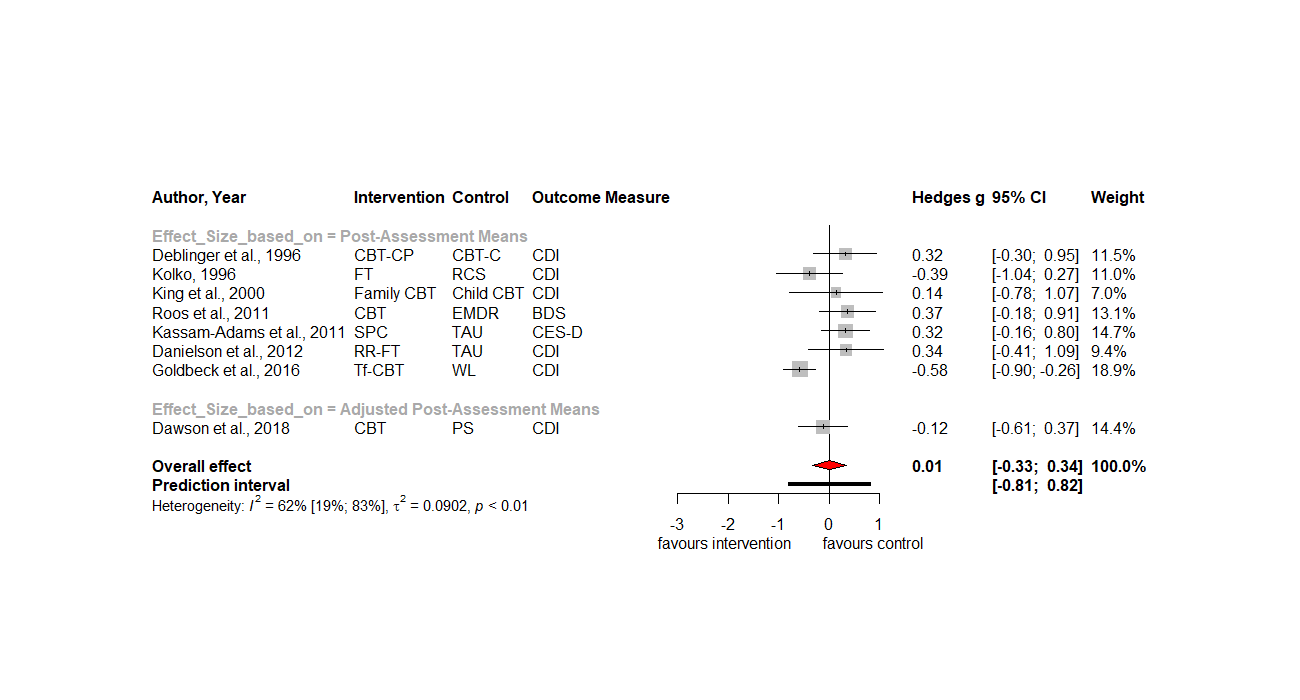


**Figure S7**

*Forest plot for child-reported depressive symptoms at six months*


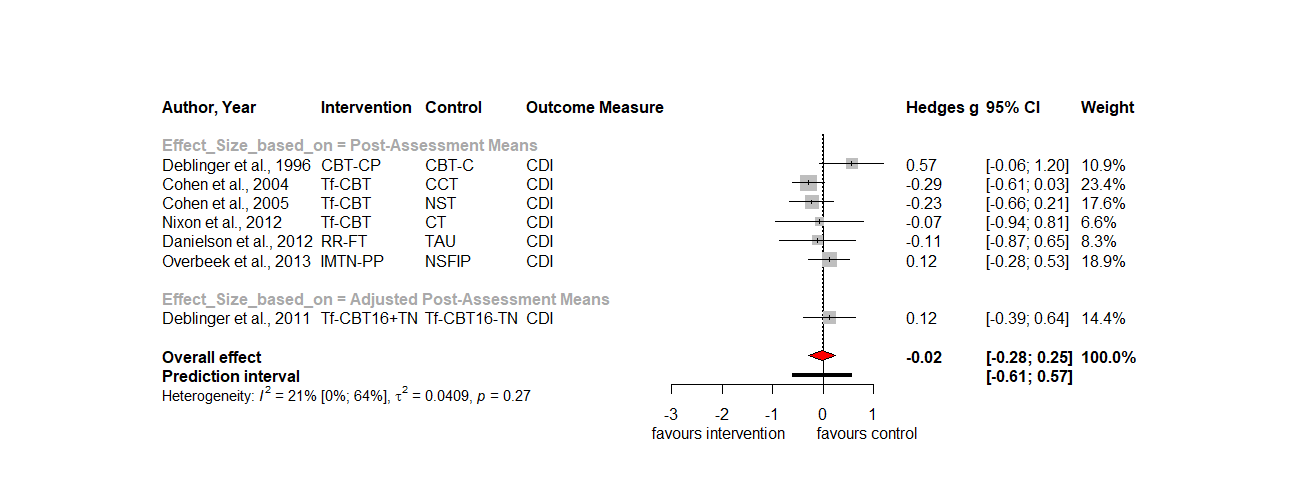


**Figure S8**

*Forest plot for child-reported depressive symptoms at twelve months*


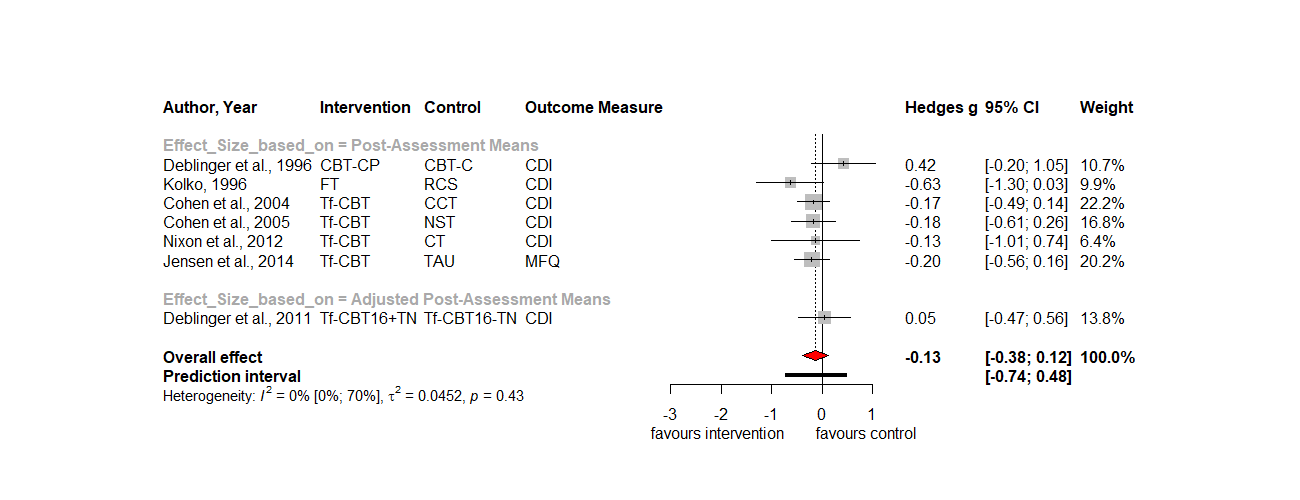


**Figure S9**

*Forest plot for child-reported anxiety symptoms at three to four months*


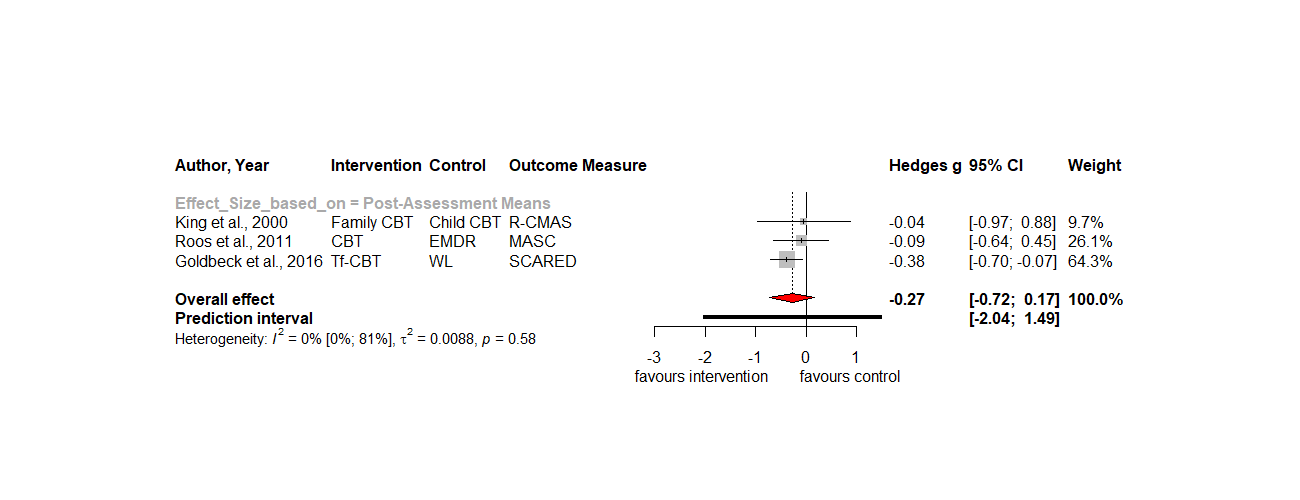


**Figure S10**

*Forest plot for child-reported anxiety symptoms at six months*


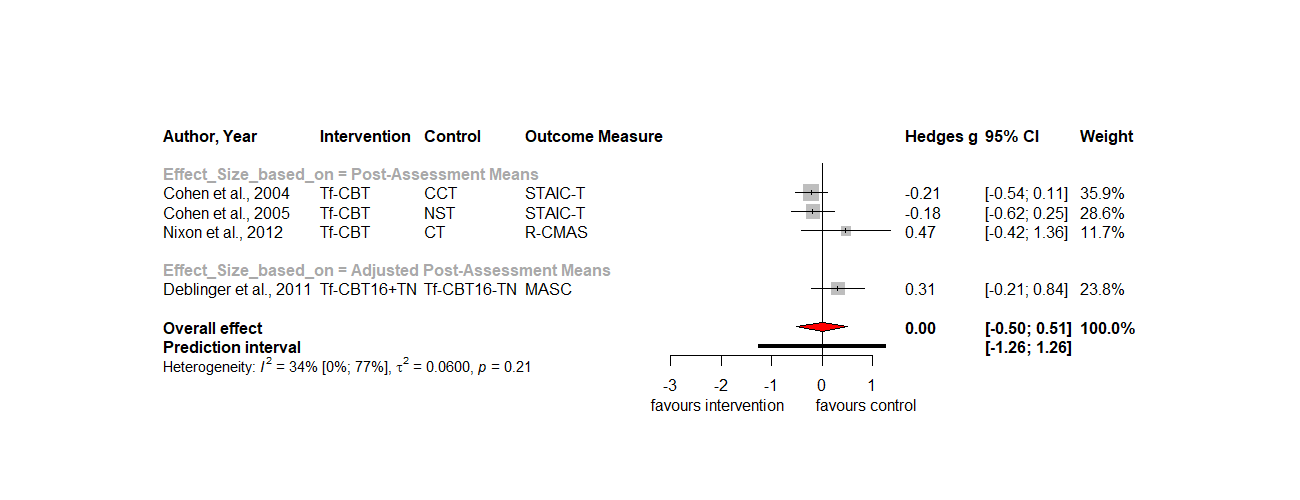


**Figure S11**

*Forest plot for child-reported anxiety symptoms at twelve months*


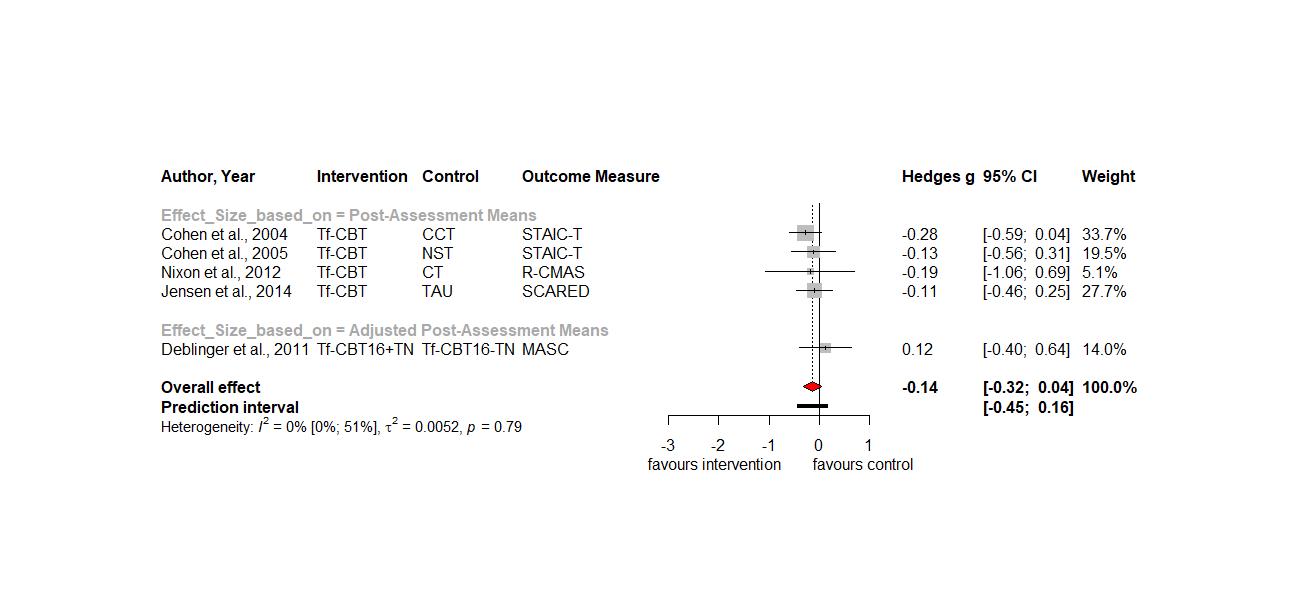


**Figure S12**

*Forest plot for child-reported internalizing symptoms at twelve months*


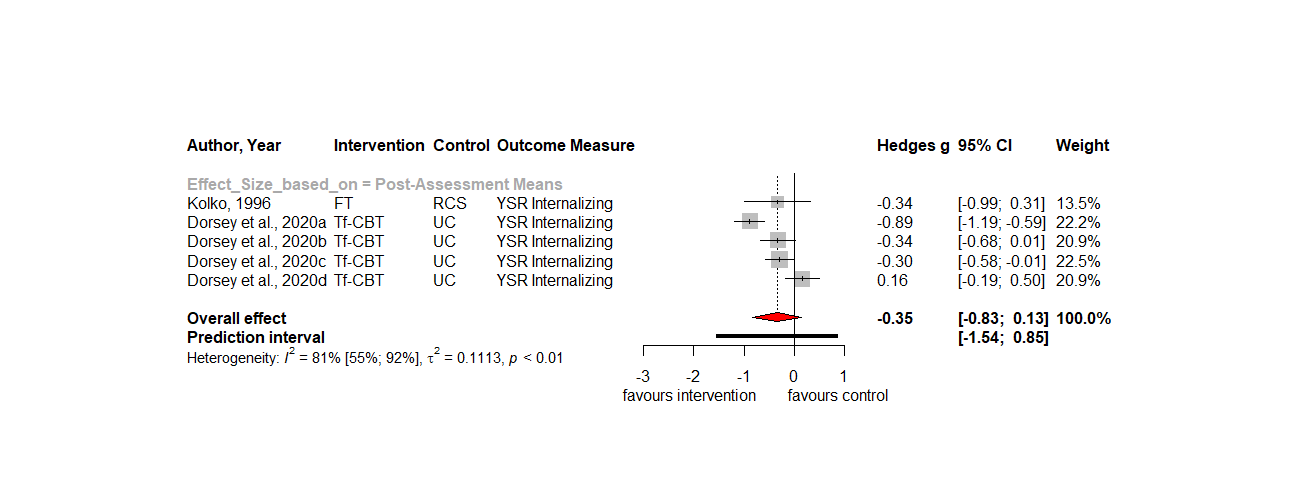


**Figure S13**

*Forest plot for internalizing symptoms at three to four months*


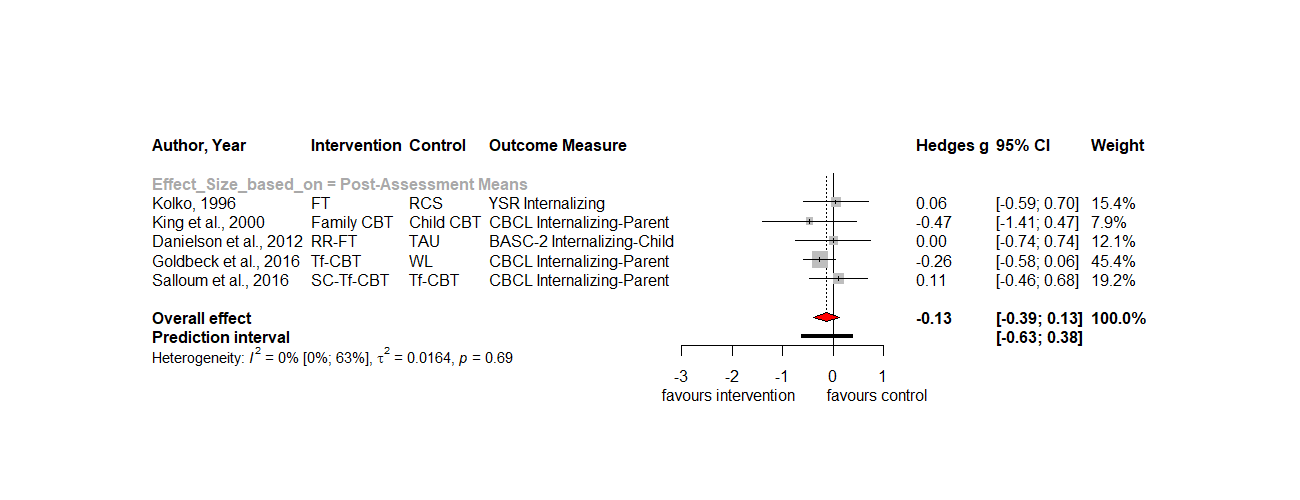


**Figure S14**

*Forest plot for internalizing symptoms at six months*


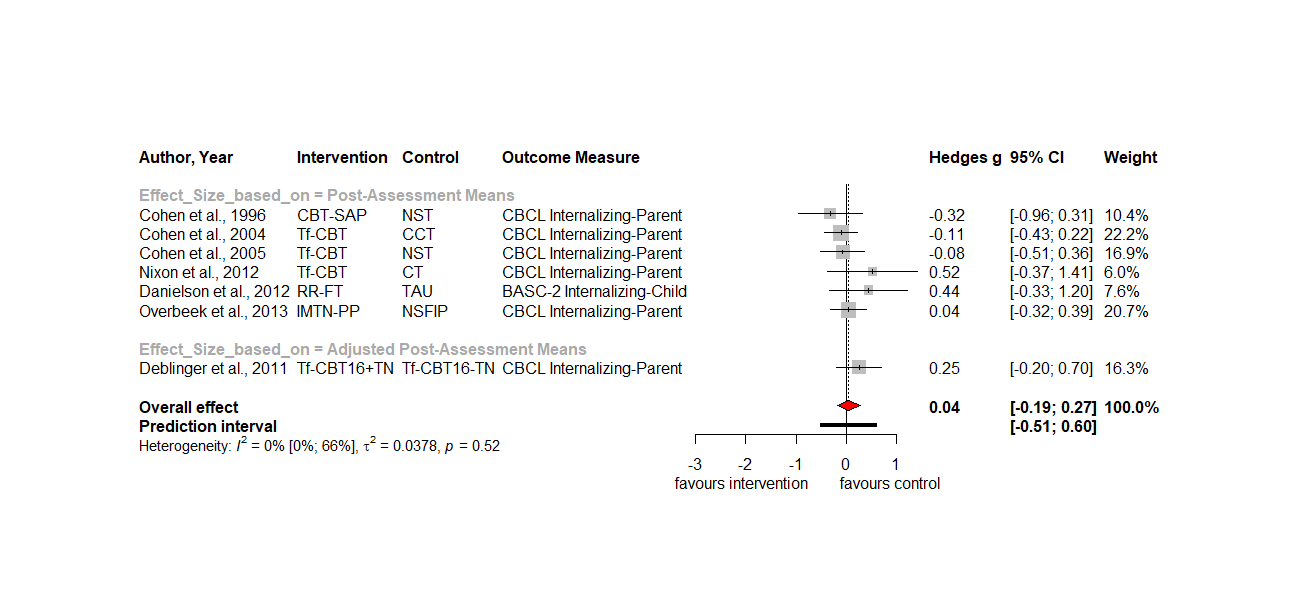


**Figure S15**

*Forest plot for parent-reported internalizing symptoms at twelve months*


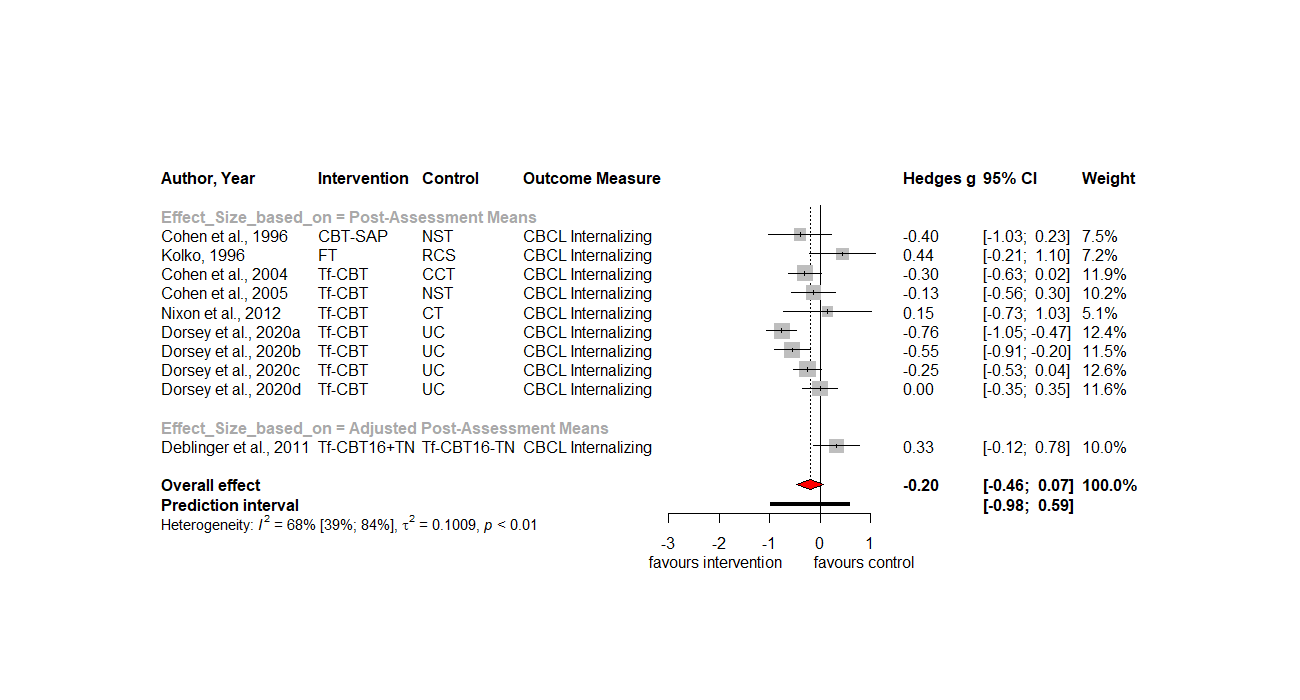


**Figure S16**

*Forest plot for child-reported externalizing symptoms at twelve months*


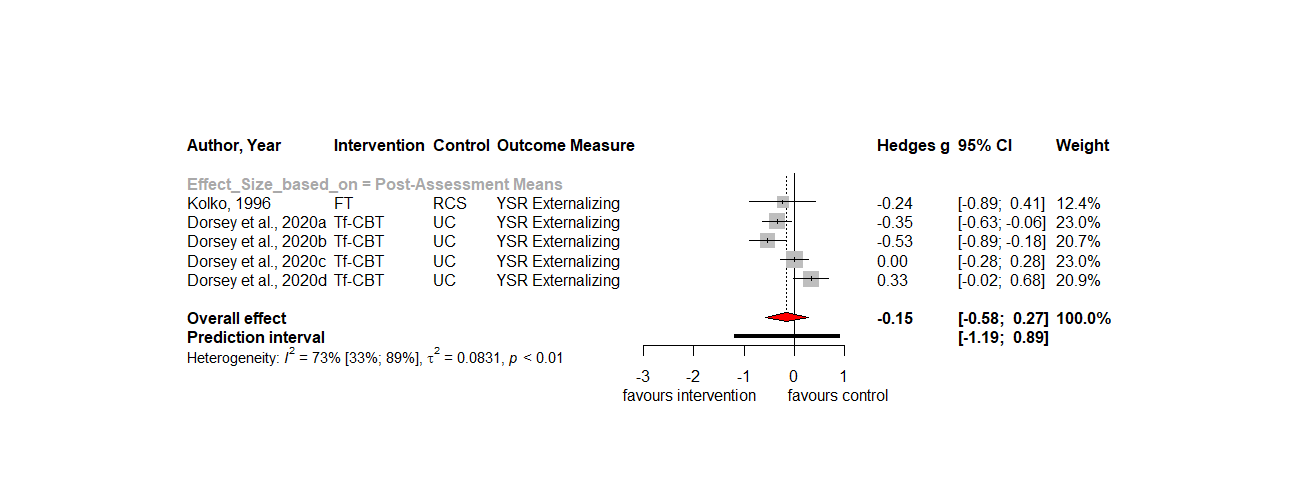


**Figure S17**

*Forest plot for externalizing symptoms at three to four months*


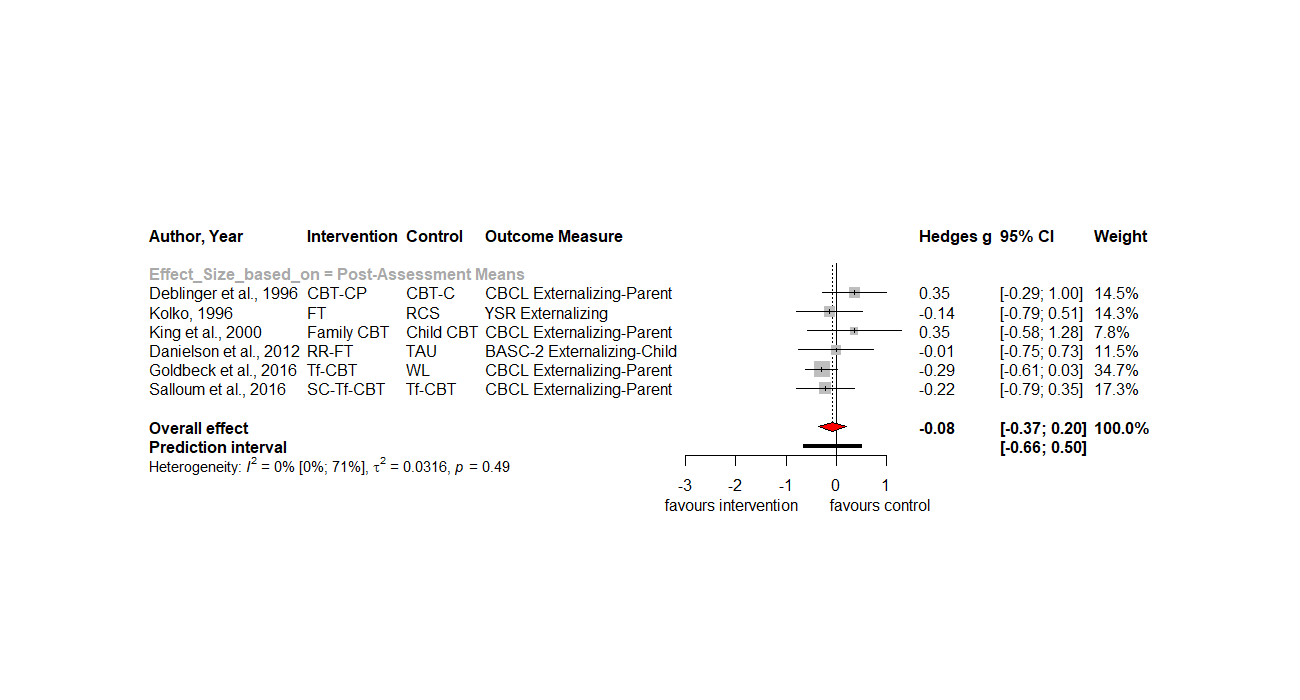


**Figure S18**

*Forest plot for externalizing symptoms at six months*


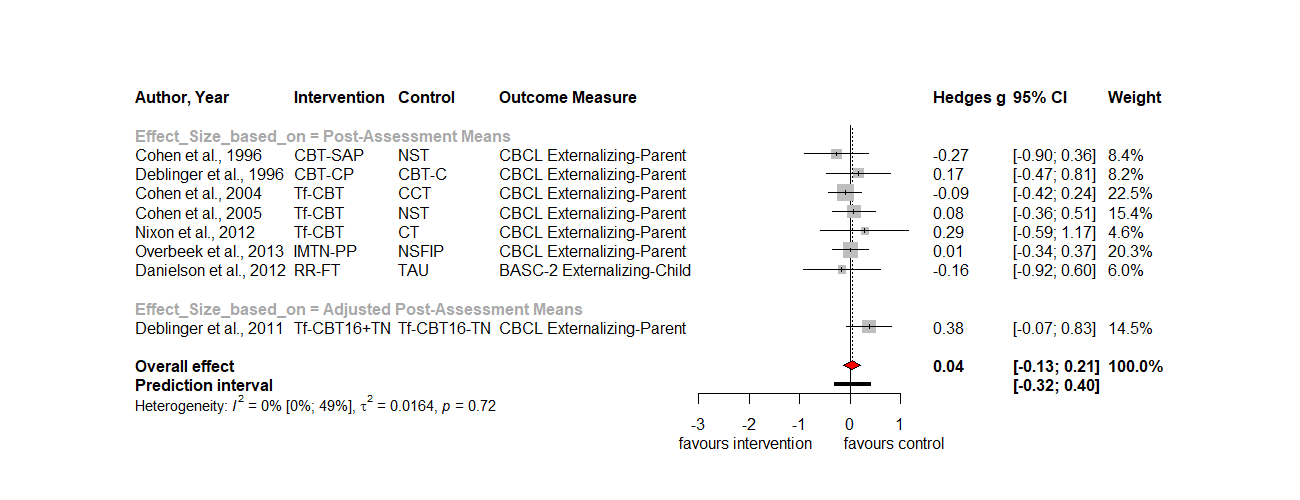


**Figure S19**

*Forest plot for parent-reported externalizing symptoms at twelve months*


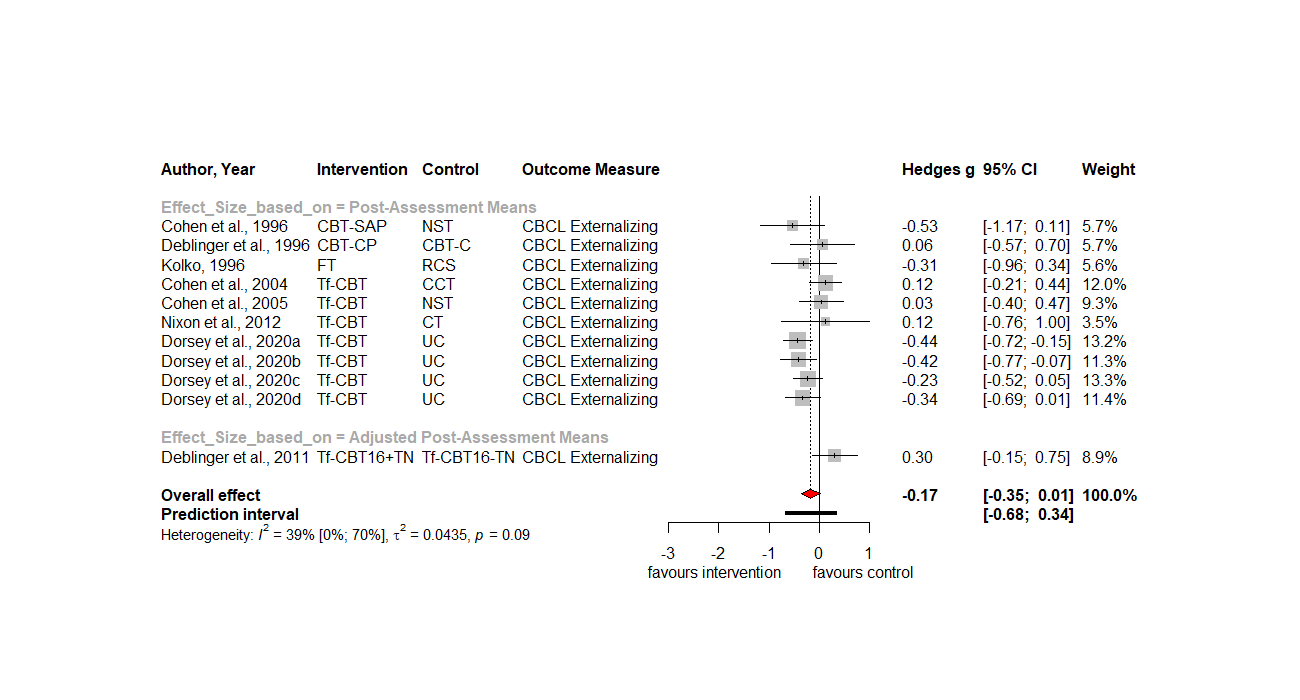


**Figure S20**

*Forest plot for behavior problems at six months*


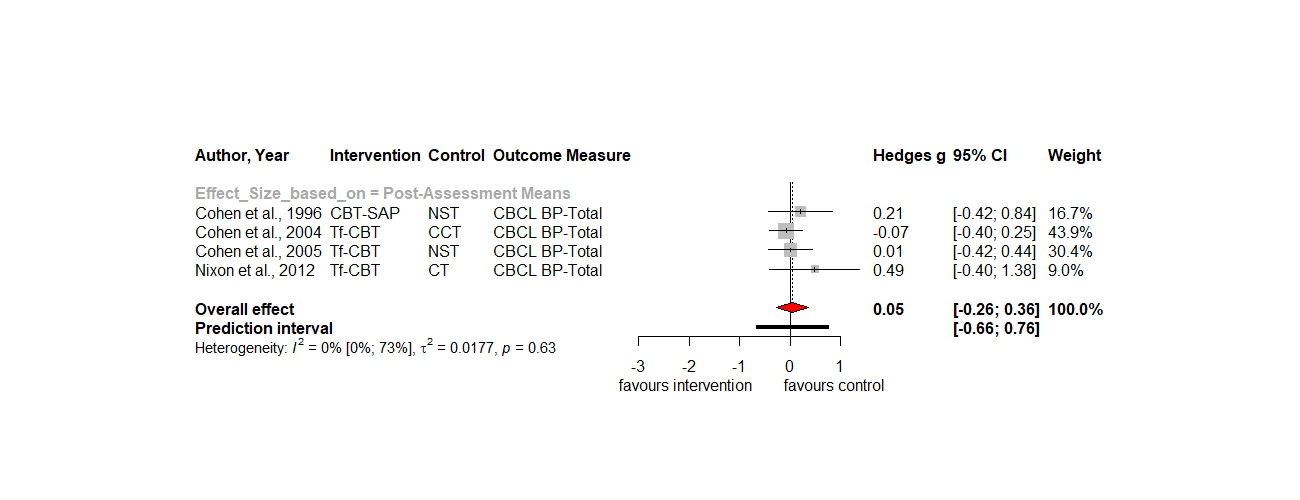


**Figure S21**

*Forest plot for behavior problems at twelve months*


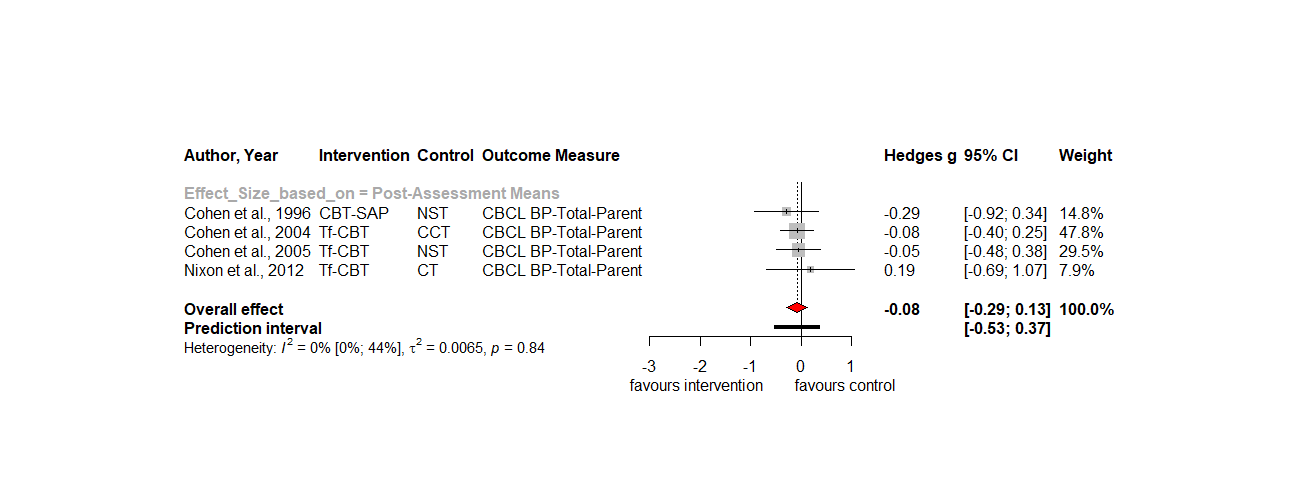

Supplement: Supplementary file 3 — Supplementary file3 (DOCX 381 kb) [file 10567_2022_415_MOESM3_ESM.docx]
