# Supplementary material for: Interventions Involving Caregivers for Children and Adolescents Following Traumatic Events: A Systematic Review and Meta-Analysis
Source: Clin Child Fam Psychol Rev. 2022 Sep 26;26(1):17–32. doi: 10.1007/s10567-022-00415-2 (PMC9879828; doi:10.1007/s10567-022-00415-2)

**Supplemental material 4**

**Figures S22-S33**

**Figure S22**

*Funnel plot for child-reported PTSD symptoms without outlier studies*


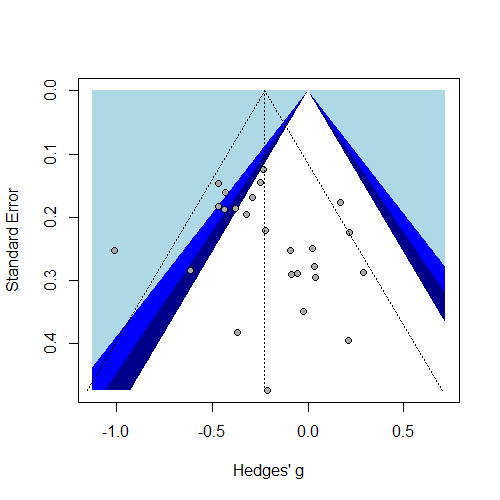


**Figure S23**

*Funnel plot for parent-reported PTSD symptoms without outlier studies*


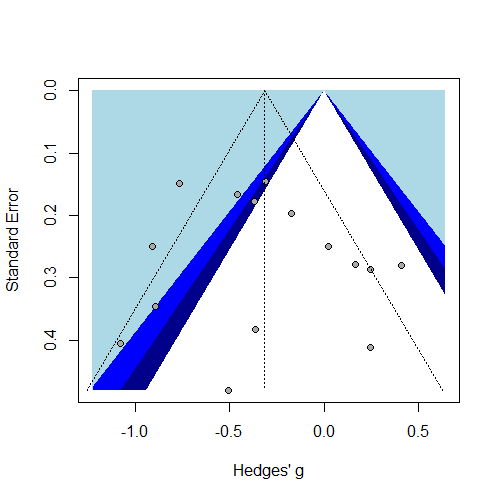


**Figure S24**

*Funnel plot for child-reported depressive symptoms without outlier studies*

*
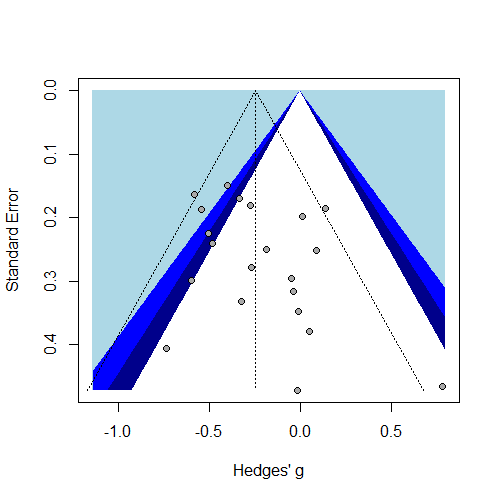
*

**Figure S25**

*Funnel plot for parent-reported depressive symptoms*


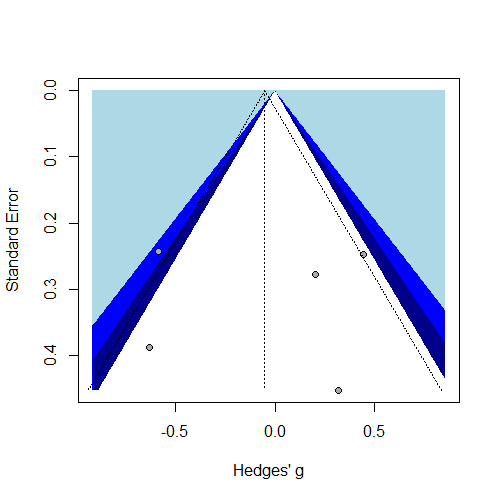


**Figure S26**

*Funnel plot for child-reported anxiety symptoms*


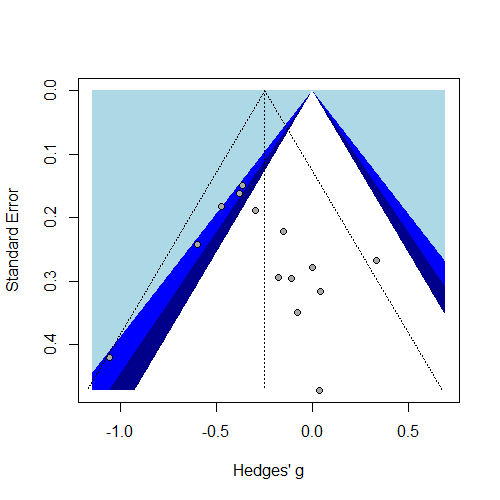


**Figure S27**

*Funnel plot for parent-reported anxiety symptoms*


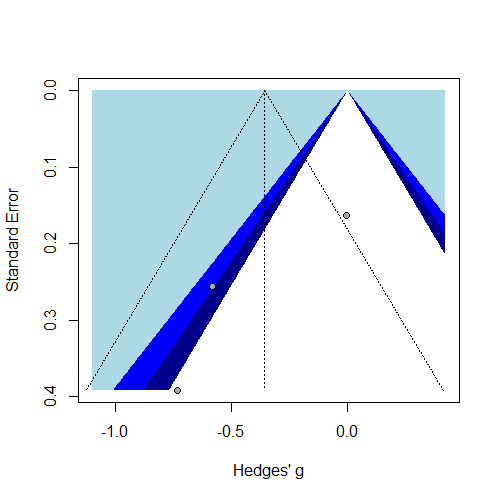


**Figure S28**

*Funnel plot for parent-reported ADHD symptoms*


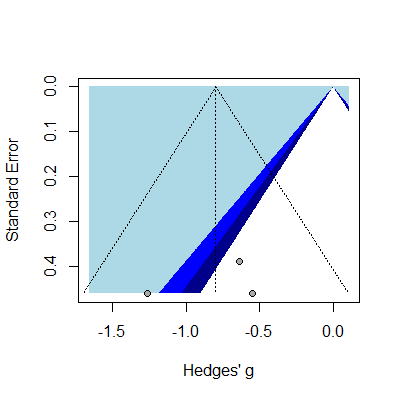


**Figure S29**

*Funnel plot for child-reported internalizing symptoms without outlier studies*


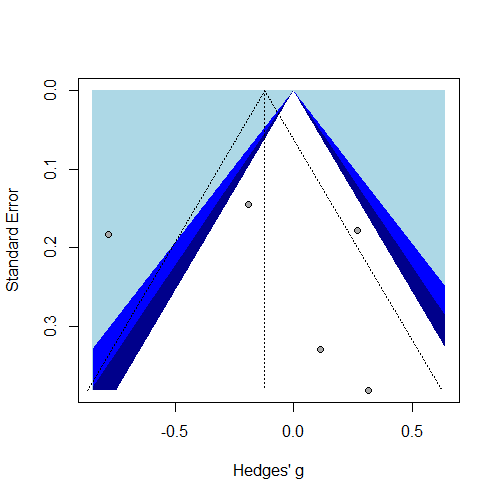


**Figure S30**

*Funnel plot for parent-reported internalizing symptoms without outlier studies*


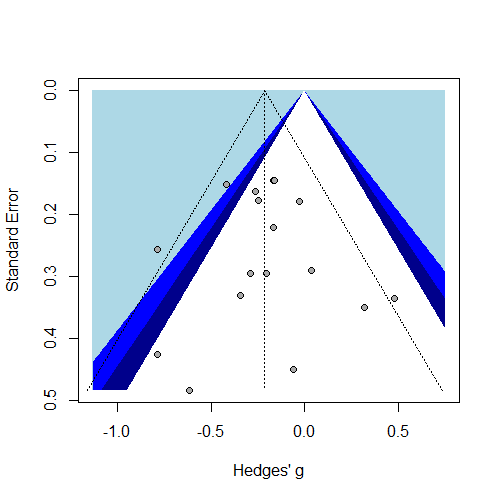


**Figure S31**

*Funnel plot for child-reported externalizing symptoms*

*
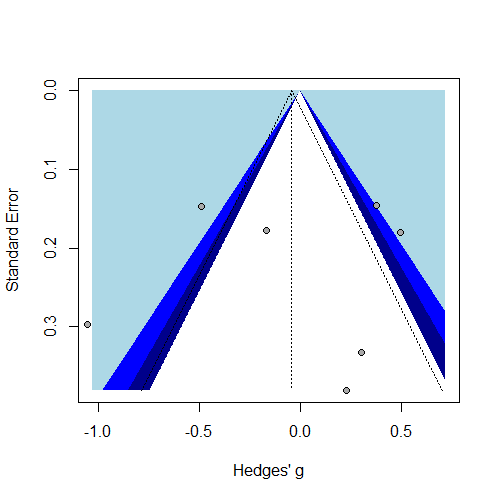
*

**Figure S32**

*Funnel plot for parent-reported externalizing symptoms*


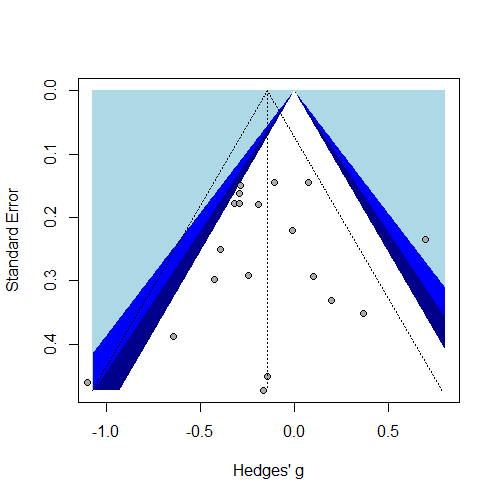


**Figure S33**

*Funnel plot for behavior problems*


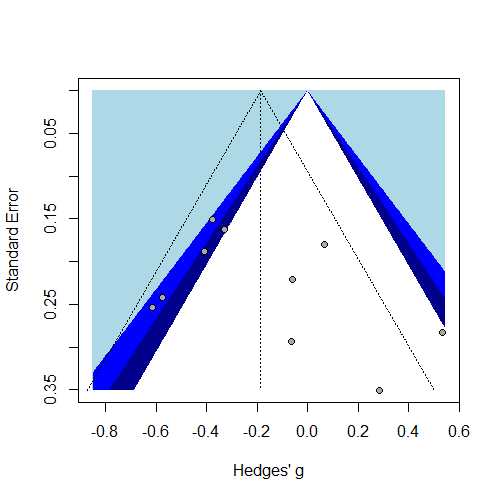

Supplement: Supplementary file 4 — Supplementary file4 (DOCX 88 kb) [file 10567_2022_415_MOESM4_ESM.docx]
